# Pd-incorporated polyoxometalate catalysts for electrochemical CO_2_ reduction†

**DOI:** 10.1039/d4sc04304a

**Published:** 2024-08-19

**Authors:** Kimitake Kawakami, Tomohiro Yabe, Fumiaki Amano, Kazuya Yamaguchi, Kosuke Suzuki

**Affiliations:** a Department of Applied Chemistry, School of Engineering, The University of Tokyo 7-3-1 Hongo Bunkyo-ku Tokyo 113-8656 Japan ksuzuki@appchem.t.u-tokyo.ac.jp; b Department of Applied Chemistry for Environment, Graduate School of Urban Environmental Sciences, Tokyo Metropolitan University 1-1 Minami-Osawa Hachioji Tokyo 192-0397 Japan

## Abstract

Polyoxometalates (POMs), representing anionic metal–oxo clusters, display diverse properties depending on their structures, constituent elements, and countercations. These characteristics position them as promising catalysts or catalyst precursors for electrochemical carbon dioxide reduction reaction (CO_2_RR). This study synthesized various salts—TBA^+^ (tetra-*n*-butylammonium), Cs^+^, Sr^2+^, and Ba^2+^—of a dipalladium-incorporated POM (Pd2, [γ-H_2_SiW_10_O_36_Pd_2_(OAc)_2_]^4−^) immobilized on a carbon support (Pd2/C). The synthesized catalysts—TBAPd2/C, CsPd2/C, SrPd2/C, and BaPd2/C—were deposited on a gas-diffusion carbon electrode, and the CO_2_RR performance was subsequently evaluated using a gas-diffusion flow electrolysis cell. Among the catalysts tested, BaPd2/C exhibited high selectivity toward carbon monoxide (CO) production (*ca.* 90%), while TBAPd2/C produced CO and hydrogen (H_2_) with moderate selectivity (*ca.* 40% for CO and *ca.* 60% for H_2_). Moreover, BaPd2/C exhibited high selectivity toward CO production over 12 h, while palladium acetate, a precursor of Pd2, showed a significant decline in CO selectivity during the CO_2_RR. Although both BaPd2/C and TBAPd2/C transformed into Pd nanoparticles and WO_*x*_ nanospecies during the CO_2_RR, the influence of countercations on their product selectivity was significant. These results highlight that POMs and their countercations can effectively modulate the catalytic performance of POM-based electrocatalysts in CO_2_RR.

## Abstract 2

We investigated the electrochemical CO_2_RR performance of a Pd-incorporated polyoxometalate ([H_2_SiW_10_O_36_Pd_2_(OAc)_2_]^4−^) possessing various countercations on a carbon electrode and found that the Ba^2+^ salt exhibited selective CO production over 12 h.

## Graphical abstract

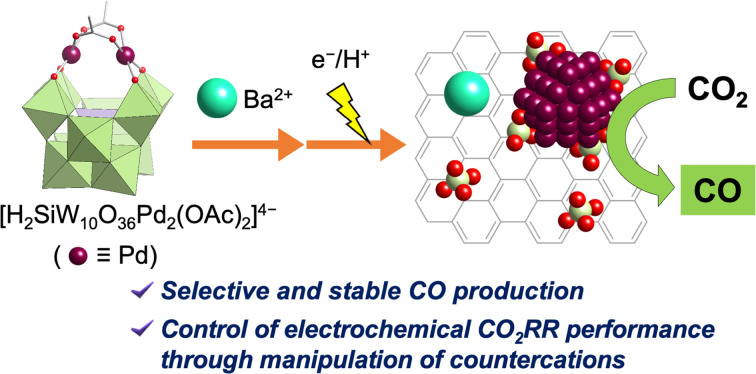

## Introduction

Anthropogenic carbon dioxide (CO_2_) emissions are widely recognized as major contributors to recent global warming, requiring prompt and effective mitigation strategies.^1^ In this context, the electrochemical CO_2_ reduction reaction (CO_2_RR) stands out as a promising solution, attracting considerable attention from both academia and industry.^2^ Utilizing multiple electrons and protons, the CO_2_RR process electrochemically converts CO_2_ into numerous valuable chemicals and fuels, such as carbon monoxide (CO), methanol, and ethylene (C_2_H_4_).^3^ Among these products, CO holds particular importance as a component of syngas, a vital feedstock for hydrocarbon synthesis *via* methanol or Fischer–Tropsch processes.^4^ Notably, the selectivity of the CO_2_RR is significantly influenced by the metals used in the electrocatalysts, with Au, Ag, and Zn demonstrating high selectivity toward CO production.^5^ In addition to these, Pd can also produce CO with high selectivity; however, its efficiency is considerably influenced by the applied potential and the state of the Pd catalyst. For instance, with increasing overpotential, the primary CO_2_RR product of Pd changes from formate (HCOO^−^) to CO, and smaller Pd nanoparticles (NPs) produce CO with greater selectivity.^6,7^ However, given the widespread use of aqueous electrolytes for the CO_2_RR, the competing hydrogen evolution reaction (HER) is inevitable,^8^ and the resulting degradation in catalytic performance emerges as a significant concern.^4^ Hence, developing robust CO_2_RR catalysts capable of selectively producing specific products is critical.

Previous studies have extensively investigated the effects of alkali metal cations in electrolytes on the CO_2_RR. Based on their findings, these cations have been widely recognized to play critical roles in driving the CO_2_RR,^9^ with larger cations such as K^+^ and Cs^+^ demonstrating greater productivity for CO, HCOO^−^, and C_2_H_4_ compared to smaller cations such as Li^+^.^9–19^ Conversely, studies conducted by Schizodimou and Kyriacou have demonstrated higher CO_2_RR activity for electrolytes containing multivalent cations, particularly at low overpotentials.^20^ Meanwhile, other reports have suggested that multivalent cations, such as alkaline earth metal cations, enhance the partial current density of CO.^14^ Notably, electrolytes with small amounts of Ba^2+^ have been observed to reduce HER activity while increasing CO_2_RR activity at high overpotentials.^17^ Conversely, Bhargava *et al.* have reported that electrolytes containing substantial amounts of CaCl_2_ and BaCl_2_ compromise the CO_2_RR performance of Ag.^16^ This diminished performance is attributed to the coverage of active sites by the deposited oxides, hydroxides, and other compounds of alkaline earth metals. Beyond these factors, the practical application of alkaline earth metal salts as electrolytes is further constrained by their lower solubility in water,^12^ often necessitating excessive quantities for effective use, and their higher cost compared to corresponding alkali metal salts. Consequently, developing systems that minimize the use of alkaline earth metal salts while demonstrating optimal CO_2_RR performance is essential.

Polyoxometalates (POMs), representing anionic metal–oxo clusters comprising multiple {MO_*x*_} units (M = W, Mo, V, *etc.*), exhibit diverse properties depending on their structures and constituent elements.^21–32^ These attributes position POMs as promising catalysts and catalyst precursors for various electrochemical and photochemical reactions including CO_2_RR.^30,33–38^ Among the known varieties of POMs, particularly noteworthy are lacunary POMs, which lack one or more {MO_*x*_} units and possess reactive oxygen atoms, thus functioning as inorganic multidentate ligands.^39,40^ Our research group has pioneered techniques for synthesizing well-defined multinuclear metal-incorporated POMs by reacting tetra-*n*-butylammonium (TBA^+^) salts of lacunary POMs with metal ions in organic solvents.^40–43^ These metal-incorporated POMs have been demonstrated to serve as promising catalysts^44–48^ or catalyst precursors^49^ for various reactions. For instance, we utilized a TBA^+^ salt of a diiron-incorporated POM (TBAFe2, TBA_8_[H_4_(SiW_10_O_36_)_2_Fe_2_O]) supported on silica (TBAFe2/SiO_2_) as a catalyst precursor for methane oxidation, selectively obtaining formaldehyde and CO.^49^ Interestingly, tungsten-oxide nanoclusters derived from TBAFe2 protected *in situ*-formed FeO_*x*_ subnanoclusters, thereby avoiding catalyst deactivation and product oxidation. Thus, metal-incorporated POMs hold immense potential for developing robust CO_2_RR catalysts capable of synthesizing specific products with high selectivity.

Notably, the countercations of POMs not only balance their anionic charges but also substantially influence their solubility and physicochemical properties.^50,51^ Typically, W-, Mo-, and V-based POMs exhibit reduced solubility in water when larger countercations such as Cs^+^ and Ba^2+^ are incorporated. Leveraging this characteristic, Blasco-Ahicart *et al.* used Cs^+^ and Ba^2+^ salts of nonacobalt-incorporated POMs as heterogeneous electrocatalysts for the oxygen evolution reaction in a 1 M H_2_SO_4_ aqueous solution.^52^ Thus, we envisaged that selecting appropriate countercations of POMs holds significant promise for developing robust and selective catalysts for the CO_2_RR.

**Fig. 1:**
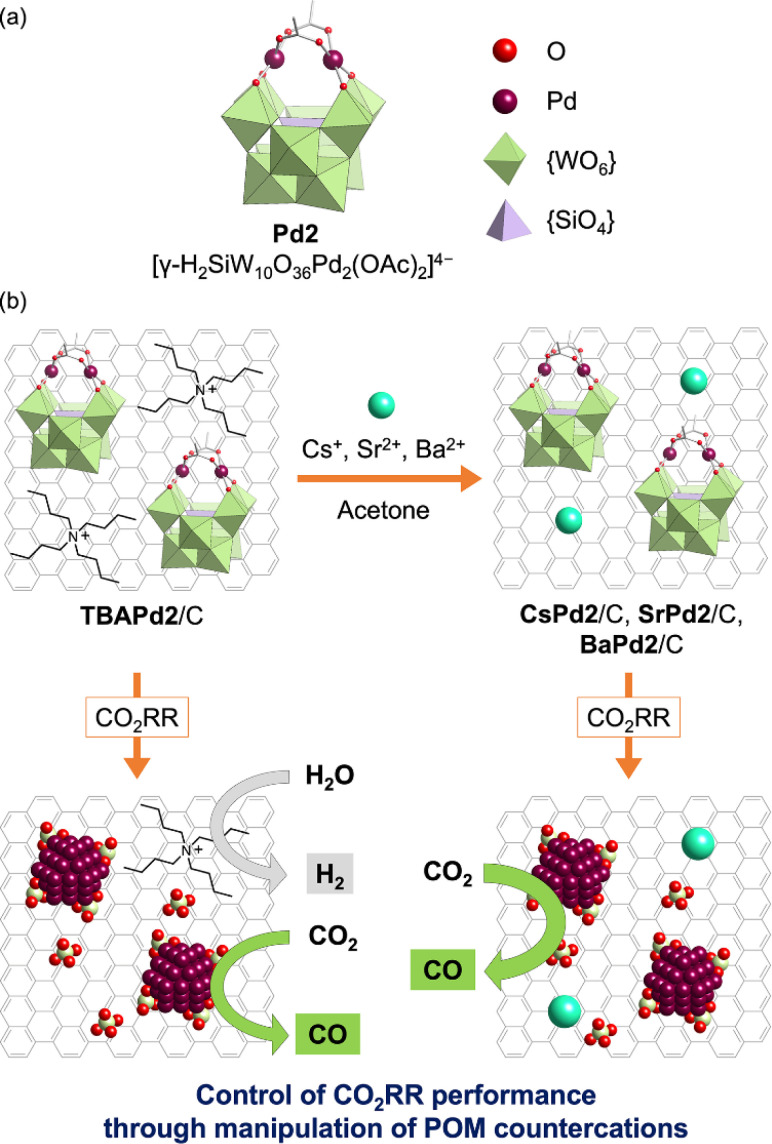
Preparation of POM-based electrocatalysts and their application in electrochemical CO_2_RR. (a) Structure of Pd2 ([γ-H_2_SiW_10_O_36_Pd_2_(OAc)_2_]^4−^). (b) Schematic depicting the preparation procedure of CsPd2/C, SrPd2/C, and BaPd2/C through manipulation of countercations of TBAPd2/C and their applications in electrochemical CO_2_RR.

Building upon these insights, this study is the first to investigate the CO_2_RR performance of Pd-incorporated POMs and the effect of their countercations. Specifically, a TBA^+^ salt of a dipalladium-incorporated POM (Pd2, [γ-H_2_SiW_10_O_36_Pd_2_(OAc)_2_]^4−^, Fig. 1a)^44^ immobilized on a carbon support (TBAPd2/C) was first synthesized. Subsequently, to explore the influence of different countercations, various analogs—CsPd2/C, SrPd2/C, and BaPd2/C—were prepared by exchanging TBA^+^ with alkali metal or alkaline earth metal cations (Cs^+^, Sr^2+^, or Ba^2+^) in an organic solvent (Fig. 1b). These catalysts were then deposited on a gas-diffusion carbon electrode, and CO_2_RR was conducted using a gas-diffusion flow electrolysis cell containing a 1 M potassium bicarbonate (KHCO_3_) aqueous solution. Our results revealed that BaPd2/C exhibited the highest selectivity toward CO production (*ca.* 90%) and maintained its catalytic performance over 12 h. Conversely, TBAPd2/C produced CO and hydrogen (H_2_) with moderate selectivity (*ca.* 40% for CO and *ca.* 60% for H_2_) (Fig. 1b). Subsequent analyses of the electrodes after participating in the CO_2_RR revealed the transformation of both BaPd2/C and TBAPd2/C into Pd NPs and WO_*x*_ nanospecies. Furthermore, Pd acetate (Pd(OAc)_2_), a precursor of Pd2, showed a significant decline in CO selectivity during the CO_2_RR. These results reveal the significant influence of POMs and their countercations on the performance of POM-based electrocatalysts for the CO_2_RR.

## Results and discussion

### Preparation and characterization of catalysts

Initially, TBAPd2 (Fig. 1a)^44^ was immobilized on a carbon support by mixing an acetone solution of TBAPd2 with an ethyl acetate suspension of the carbon support, yielding TBAPd2/C (Fig. 1b).^53^ Subsequently, cation exchange was performed by immersing TBAPd2/C in an acetone solution containing trifluoromethanesulfonate salts of Cs^+^, Sr^2+^, and Ba^2+^ to obtain CsPd2/C, SrPd2/C, and BaPd2/C, respectively (Fig. 1b, see ESI† for the detailed procedure).^54^ This approach of conducting cation exchange on a carbon support was preferred over direct immobilization of Cs^+^, Sr^2+^, and Ba^2+^ salts of Pd2 (*i.e.*, CsPd2, SrPd2, and BaPd2) owing to the low solubility of the resulting POMs in water and organic solvents,^50^ which complicates their direct immobilization.

**Fig. 2:**
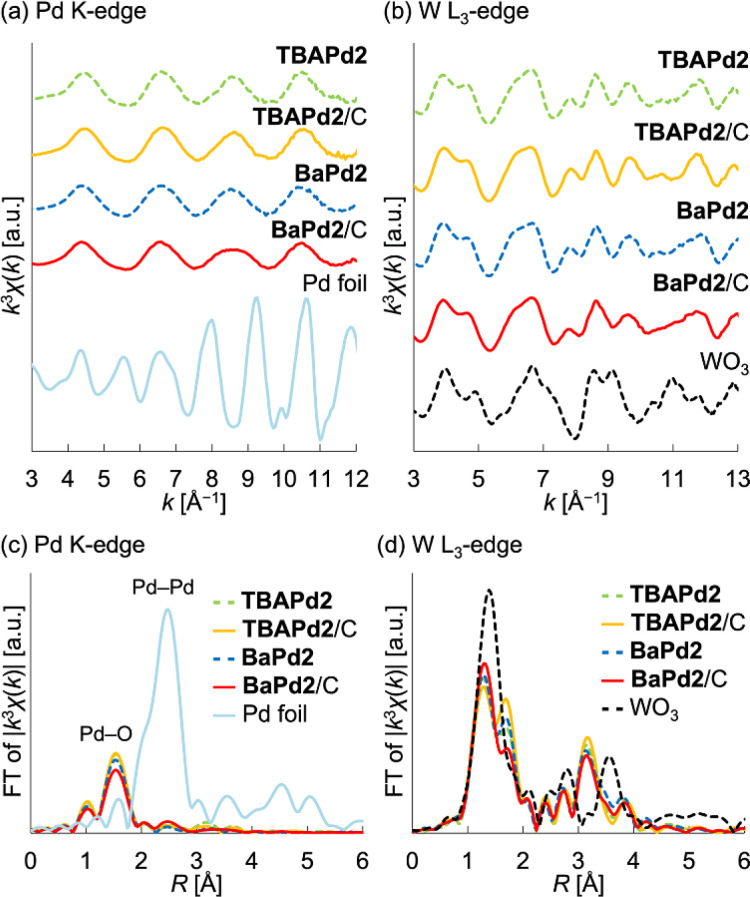
XAFS characterizations of TBAPd2/C and BaPd2/C. (a) *k*^3^-weighted Pd K-edge EXAFS oscillations (*k* = 3–12 Å^−1^). (b) *k*^3^-weighted W L_3_-edge EXAFS oscillations (*k* = 3–13 Å^−1^). (c) Fourier-transformed Pd K-edge EXAFS spectra. (d) Fourier-transformed W L_3_-edge EXAFS spectra.

Following synthesis, the prepared catalysts were characterized through X-ray absorption fine structure (XAFS) measurements. Notably, the Pd K-edge and W L_3_-edge extended X-ray absorption fine structure (EXAFS) oscillation patterns, as well as the Fourier-transformed EXAFS spectra, of TBAPd2/C closely resembled those of TBAPd2 (Fig. 2), suggesting that the anion structure of TBAPd2 remained intact upon immobilization on the carbon support. Subsequently, the EXAFS spectra of the cation-exchanged samples were similarly examined. As depicted in Fig. 2a, c, S1a and Table S1,† although the Fourier-transformed Pd K-edge EXAFS spectrum of BaPd2/C presented a lower intensity of scattering from the Pd–O bond compared to that of TBAPd2/C, the Pd K-edge EXAFS oscillation pattern of BaPd2/C closely resembled that of TBAPd2/C. Similarly, the W L_3_-edge EXAFS oscillation pattern of BaPd2/C closely matched that of TBAPd2/C (Fig. 2b). However, compared to the Fourier-transformed W L_3_-edge EXAFS spectrum of TBAPd2/C, that of BaPd2/C displayed increased scattering intensity from the terminal W=O bond (peak around 1.3 Å)^55^ and decreased scattering intensity from the W–O(–W) bond (bonds between tungsten atoms and the bridging oxygen atom, with a peak around 1.7 Å)^55^ (Fig. 2d, S1b and Table S1†). These findings suggest that although the coordination environment of Pd and W partially changed during the cation exchange process, the fundamental structure of Pd2 was intrinsically maintained.

Based on these XAFS findings, we further investigated the structure of Pd2 during cation exchange by subjecting TBAPd2/C and BaPd2/C to infrared (IR) measurements. However, IR peaks originating from the POMs were difficult to discern owing to interference from the carbon support. To resolve this, we subsequently analyzed the IR spectra of TBAPd2 and BaPd2 without their carbon supports (Fig. S2†). Specifically, BaPd2 was synthesized through the cation exchange of TBAPd2 with Ba^2+^. Interestingly, the IR spectrum of BaPd2 displayed no peak corresponding to TBA^+^ in the range of 2800–3000 cm^−1^, indicating successful cation exchange. Furthermore, the IR spectrum of BaPd2 in the range of 300–1000 cm^−1^ closely resembled that of TBAPd2. Given that this region is characteristic of Keggin-type POMs,^56^ the cation exchange was deemed to not adversely affect the anion structure of Pd2.

Our next investigation focused on the elemental analysis of the prepared catalysts. As indicated in Table S2,† the ratio of Pd to W remained nearly constant at 2 : 10 across all samples, consistent with the composition of TBAPd2, containing two Pd atoms and ten W atoms. Hence, this finding further corroborates the results of the EXAFS analysis, indicating that the structure of Pd2 is largely preserved during immobilization on the carbon support and cation exchange. Furthermore, our elemental analysis revealed that the numbers of Cs, Sr, and Ba species in CsPd2/C, SrPd2/C, and BaPd2/C were approximately four, two, and two, respectively. Given that Pd2 is a tetravalent anion ([γ-H_2_SiW_10_O_36_Pd_2_(OAc)_2_]^4−^) and Cs^+^, Sr^2+^, and Ba^2+^ are monovalent, divalent, and divalent, respectively, these results suggest that most of the TBA^+^ was exchanged with the corresponding alkali metal and alkaline earth metal cations.

### CO_2_RR study

**Fig. 3:**
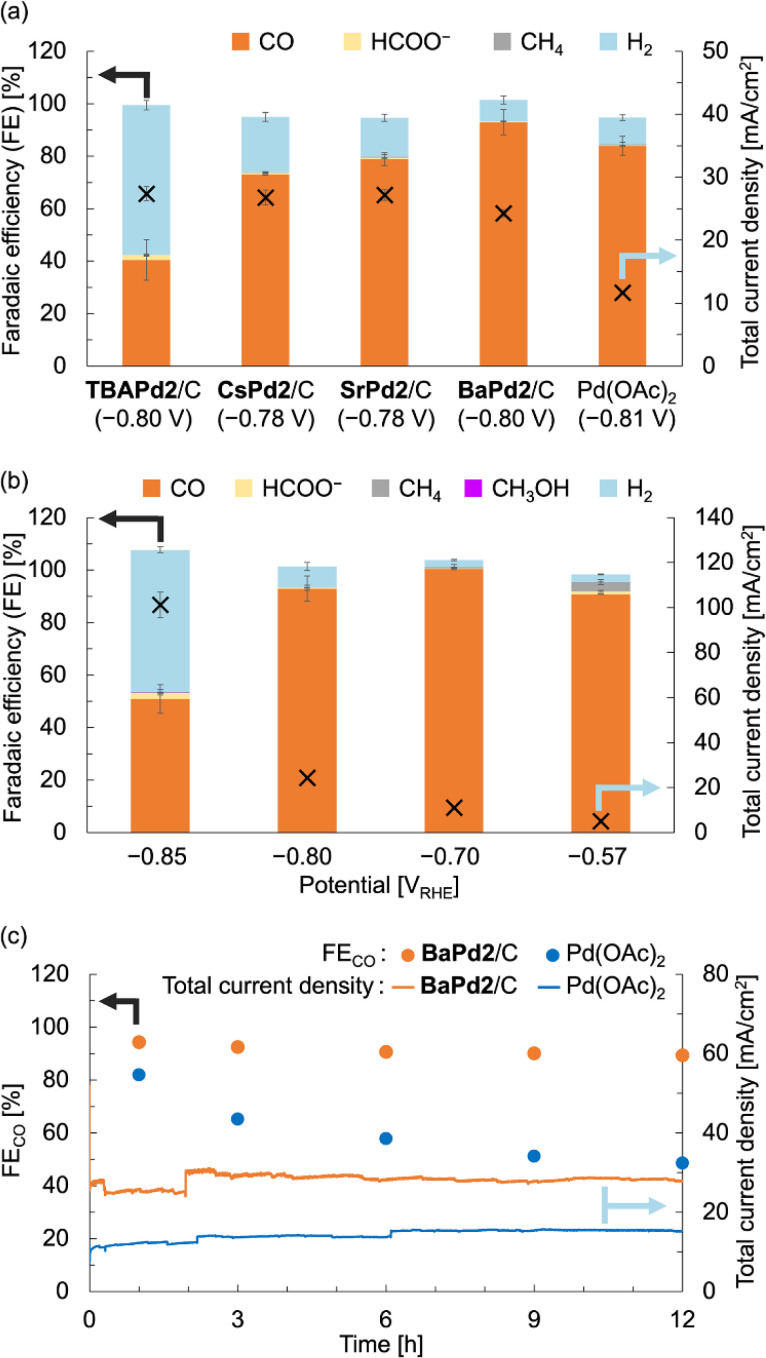
CO_2_RR performance of BaPd2/C-modified electrode and other electrodes in a 1 M KHCO_3_ aqueous solution. (a) FEs for all CO_2_RR products (left axis) and total current densities (right axis) of TBAPd2/C-, CsPd2/C-, SrPd2/C-, BaPd2/C-, and Pd(OAc)_2_-modified electrodes determined through constant potential electrolysis at −0.8 V_RHE_ for 1 h. (b) FEs for all CO_2_RR products (left axis) and total current density (right axis) of BaPd2/C-modified electrode determined through constant potential electrolysis at different potentials for 1 h. (c) FE_CO_ (left axis) and total current density (right axis) of BaPd2/C-modified electrode (−0.75 V_RHE_, orange) and Pd(OAc)_2_-modified electrode (−0.73 V_RHE_, light blue) for 12 h. Error bars represent standard deviations calculated from two independent measurements. Symbols X in (a and b) denote the total current density (right vertical axes). All potentials were IR-corrected (see ESI†).

After characterizing the prepared catalysts, we evaluated their catalytic performance in the electrochemical CO_2_RR using a gas-diffusion flow electrolysis cell (Fig. S3†).^57,58^ The working electrodes for the CO_2_RR were fabricated by depositing TBAPd2/C, CsPd2/C, SrPd2/C, and BaPd2/C onto a gas-diffusion carbon electrode (Sigracet 39 BB, see ESI† for the detailed method). These electrodes were then put in the gas-diffusion flow electrolysis cell, and their catalytic performance was assessed *via* constant potential electrolysis at approximately −0.8 V_RHE_ for 1 h, using a 1 M KHCO_3_ aqueous solution as the electrolyte. As illustrated in Fig. 3a and S4 and Table S3,†TBAPd2/C exhibited a faradaic efficiency for CO production (FE_CO_) of 40.5 ± 7.7% and a total current density of 27.4 ± 1.1 mA cm^−2^. Conversely, CsPd2/C, SrPd2/C, and BaPd2/C displayed FE_CO_ values of 72.9 ± 0.1%, 78.9 ± 2.5%, and 92.9 ± 4.8%, respectively. Furthermore, the total current densities for CsPd2/C, SrPd2/C, and BaPd2/C were 26.8 ± 1.2, 27.2 ± 0.8, and 24.3 ± 0.6 mA cm^−2^, respectively. These findings indicate significant enhancements in CO_2_RR performance following cation exchange from TBA^+^ to Cs^+^, Sr^2+^, and Ba^2+^, with BaPd2/C demonstrating the highest efficiency. Collectively, these results underscore the critical significance of countercation identity in catalytic performance, as well as the superior efficiency of BaPd2/C for the CO_2_RR. Encouraged by these outcomes, we subsequently compared the catalytic performance of BaPd2/C with that of Pd(OAc)_2_, a precursor of Pd2, deposited on a gas-diffusion carbon electrode in a similar manner to the other catalysts, at the same Pd content (Fig. 3a). The results revealed that Pd(OAc)_2_ exhibited lower FE_CO_ (83.9 ± 3.6%) and lower total current density (11.66 ± 0.04 mA cm^−2^) compared to BaPd2/C (FE_CO_ 92.9 ± 4.8%, 24.3 ± 0.6 mA cm^−2^). Although various reports have addressed CO_2_RR using POM-containing electrodes in aqueous electrolytes, there have been no reports on CO_2_RR using Pd-incorporated POMs or the effect of the countercations of POMs on CO_2_RR (Table S8†).

Subsequently, CO_2_RR experiments were performed utilizing the BaPd2/C-modified electrode at various applied potentials to elucidate the potential dependence of its catalytic performance. Although the CO_2_RR experiments at −0.70 and −0.57 V_RHE_ exhibited reduced total current densities compared to the reaction at −0.80 V_RHE_, CO was exclusively produced at each potential (Fig. 3b and Table S4†). However, reducing the potential to −0.85 V_RHE_ resulted in a decrease in FE_CO_ and an increase in a faradaic efficiency for H_2_ production (FE_H2_) to nearly 50%. We also confirmed that CO and HCOO^−^ were not produced in reactions using an electrode without Pd2 deposition or in reactions conducted in an Ar atmosphere (Table S5†).

To assess the long-term stability of the catalyst, a CO_2_RR experiment employing BaPd2/C as the catalyst was performed over 12 h at an applied potential of −0.75 V_RHE_ (Fig. 3c and Table S6†). Note that due to the instability of Sigracet 39 BB in prolonged CO_2_RR, AvCarb P75T was used as a carbon electrode to prepare a BaPd2/C-modified electrode in this experiment. As depicted in Fig. 3c, although the total current density of the catalyst fluctuated during the reaction, its selectivity toward CO production was maintained consistently throughout the experiment. In contrast, a 12 h CO_2_RR experiment using Pd(OAc)_2_ as the catalyst exhibited a significant decline in FE_CO_ during the reaction, accompanied by an increase in FE_H2_ (Fig. 3c and Table S7†). The total current density in this case was stable around 14 mA cm^−2^ throughout the reaction, approximately half that of BaPd2/C. These results highlight the significant influence of POMs for the CO_2_RR performance.

### Characterization of a BaPd2/C-modified electrode after the CO_2_RR

**Fig. 4:**
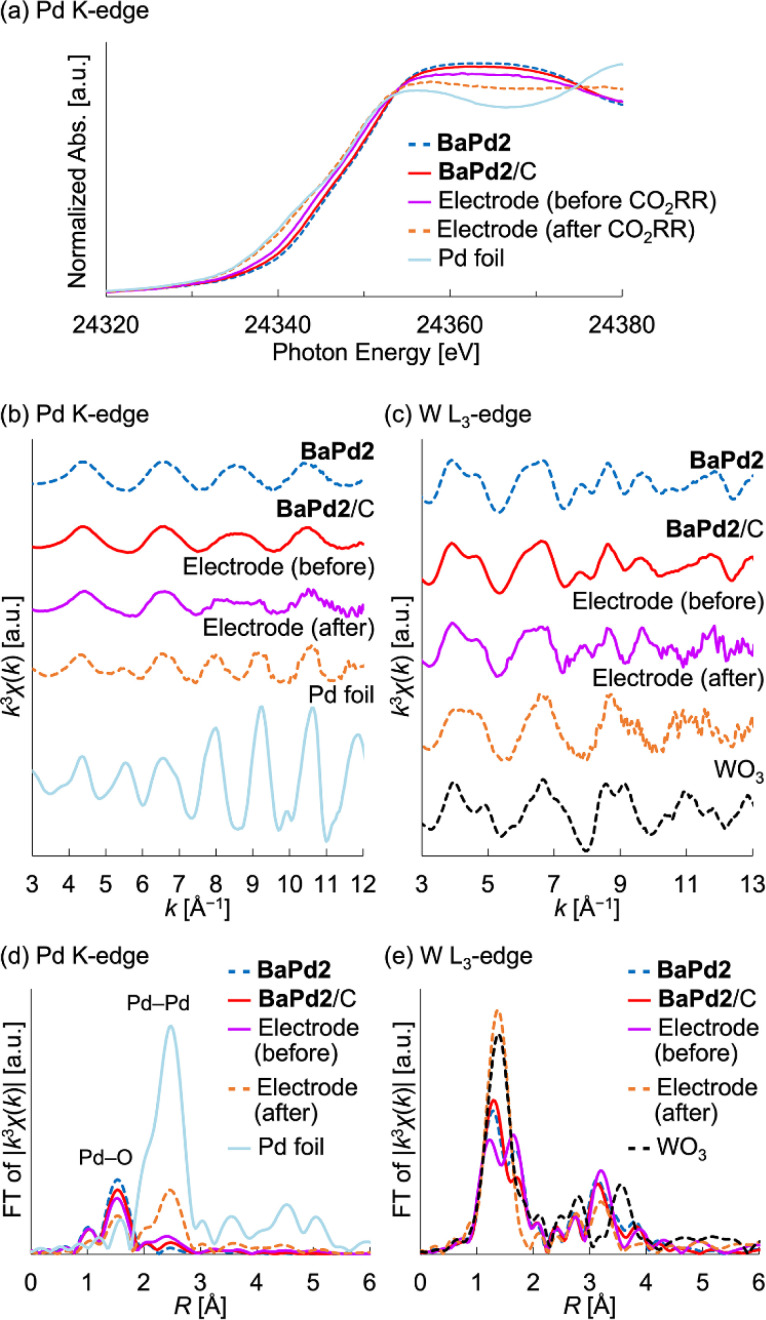
XAFS characterization of the BaPd2/C-modified electrode obtained after a 1 h CO_2_RR. (a) Pd K-edge XANES spectra. (b) *k*^3^-weighted Pd K-edge EXAFS oscillation pattern (*k* = 3–12 Å^−1^). (c) *k*^3^-weighted W L_3_-edge EXAFS oscillation pattern (*k* = 3–13 Å^−1^). (d) Fourier-transformed Pd K-edge EXAFS spectra. (e) Fourier-transformed W L_3_-edge EXAFS spectra.

Based on the results of the CO_2_RR experiments, we performed XAFS measurements to probe the structure and electronic state of a BaPd2/C-modified electrode obtained after a 1 h CO_2_RR at −0.80 V_RHE_. To investigate the electronic state of the Pd species, we measured the Pd K-edge X-ray absorption near-edge structure (XANES) spectra of the electrode before and after the CO_2_RR (Fig. 4a). Notably, the XANES spectrum of the electrode before the reaction was intermediate to the corresponding spectra of a Pd foil and BaPd2/C. Conversely, the XANES spectrum of the electrode after the reaction closely resembled that of the Pd foil. These findings indicate that Pd^2+^ underwent partial reduction during electrode fabrication and complete reduction to Pd^0^ during the CO_2_RR. Subsequently, to glean insights into the structure of the POM before and after the reaction, we performed Pd K-edge and W L_3_-edge EXAFS analysis (Fig. 4b–e). Notably, the Pd K-edge EXAFS oscillation pattern of the electrode after the CO_2_RR resembled that of the Pd foil but not that of BaPd2/C (Fig. 4b). Furthermore, the Fourier-transformed Pd K-edge EXAFS spectra revealed that the electrode after the CO_2_RR exhibited reduced scattering intensity from the Pd–O bond and increased scattering intensity from the Pd–Pd bond compared to that before the reaction (Fig. 4d). These results suggest the structural transformation of BaPd2 to Pd NPs during the reaction. Conversely, the W L_3_-edge EXAFS analysis of the electrode after the reaction displayed a similar yet partially distinct oscillation pattern compared to that of WO_3_ (Fig. 4c). Furthermore, compared to the Fourier-transformed W L_3_-edge EXAFS spectra of WO_3_, that of the electrode after the CO_2_RR displayed differences around the second coordination sphere (Fig. 4e).

For comparison, we also conducted XAFS measurements on a Pd(OAc)_2_-modified electrode subjected to a 1 h CO_2_RR (Fig. S5 and S6†). Notably, the Pd K-edge XANES spectrum of the electrode before the reaction was intermediate to those of the Pd foil and Pd(OAc)_2_, whereas after the CO_2_RR, it closely resembled that of the Pd foil (Fig. S5†). Similarly, the Pd K-edge EXAFS oscillation pattern of the electrode after the CO_2_RR resembled that of the Pd foil but not that of Pd(OAc)_2_ (Fig. S5†). Furthermore, the Fourier-transformed Pd K-edge EXAFS spectrum of the Pd(OAc)_2_-modified electrode after the CO_2_RR exhibited a similar intensity of scattering from the Pd–O bond but a greater intensity of scattering from the Pd–Pd bond compared to the BaPd2/C-modified electrode after the reaction (Fig. S6†). These results suggest that the Pd NPs transformed from Pd(OAc)_2_ were larger than those formed from BaPd2/C after the CO_2_RR.

**Fig. 5:**
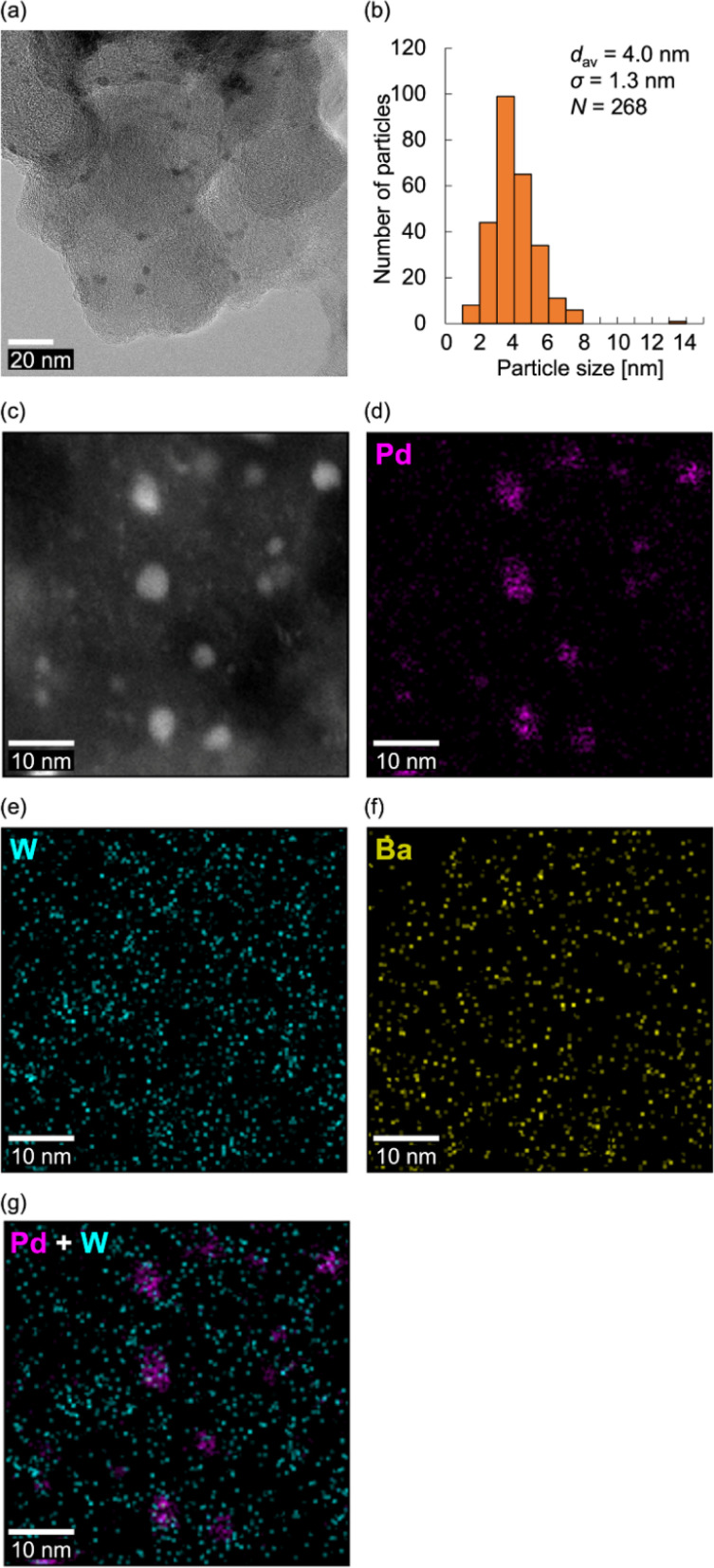
TEM and HAADF-STEM images of the BaPd2/C-modified electrode obtained after a 1 h CO_2_RR. (a) TEM image and (b) the corresponding size-distribution histogram. (c) HAADF-STEM image and (d–g) the corresponding STEM-EDS mappings (Pd, pink; W, turquoise; Ba, yellow).

Based on these findings, we subjected the BaPd2/C-modified electrode obtained after the CO_2_RR to electron microscopy observations. Notably, transmission electron microscopy (TEM) images of the BaPd2/C after the CO_2_RR revealed the formation of NPs with an average particle size of 4.0 ± 1.3 nm on the carbon support (Fig. 5a and b). Conversely, larger NPs with a broader size distribution (an average particle size of 6.0 ± 3.1 nm) were formed on the Pd(OAc)_2_-modified electrode after the CO_2_RR (Fig. S7†), consistent with the XAFS results. Subsequently, high-angle annular dark-field scanning TEM (HAADF-STEM) images (Fig. 5c) and STEM-energy-dispersive spectroscopy (EDS) mappings (Fig. 5d–g) of the BaPd2/C after the reaction revealed that most Pd NPs were surrounded by W atoms, with some W species present in locations where Pd NPs were absent. This observation also confirms the retention of Ba on the electrode after the CO_2_RR. Collectively, these results indicate that BaPd2 transformed into WO_*x*_ nanospecies and Pd NPs surrounded by WO_*x*_ nanospecies during the reaction. Adopting a similar approach, our research group previously developed various metal NPs stabilized by lacunary POMs.^59–61^ Furthermore, previous reports suggest that WO_*x*_ nanoclusters derived from diiron-incorporated POMs hinder the aggregation of *in situ*-formed FeO_*x*_ subnanoclusters.^49^ Consequently, it is plausible that WO_*x*_ nanospecies prevented the aggregation of Pd NPs in our CO_2_RR system.

### Effect of countercations on the CO_2_RR

To examine the impact of countercations on CO_2_RR performance, we characterized the TBAPd2/C-modified electrode subjected to a 1 h CO_2_RR at −0.80 V_RHE_, which exhibited poor CO_2_RR performance compared to the BaPd2/C-modified electrode (Fig. 3a and Table S3†). Notably, the Pd K-edge and W L_3_-edge EXAFS oscillation and Fourier-transformed EXAFS spectra of the TBAPd2/C-modified electrode after the CO_2_RR closely resembled those of the BaPd2/C-modified electrode after the reaction (Fig. S6 and S8†). Furthermore, the TEM image of the TBAPd2/C after the CO_2_RR revealed the formation of NPs with an average particle size of 2.9 ± 1.1 nm on the carbon support (Fig. S9a and S9b†). Similarly, the HAADF-STEM image (Fig. S9c†) and STEM-EDS mappings (Fig. S9d–9f†) of the TBAPd2/C after the CO_2_RR were nearly identical to those of the BaPd2/C after the CO_2_RR (Fig. 5). These results indicate that similar species were formed after the CO_2_RR regardless of the countercation type.

An explanation for the enhanced CO production could be related to the electronic state of the Pd species. For instance, electron-rich Pd species modified with *N*-heterocyclic carbene ligands have been reported to demonstrate superior CO_2_RR performance.^62,63^ To probe the electronic states of the Pd species, we performed CO stripping voltammetry experiments on the TBAPd2/C-, CsPd2/C-, SrPd2/C-, and BaPd2/C-modified electrodes after the CO_2_RR (Fig. S10, see ESI† for additional details). Notably, the CO stripping potential of the TBAPd2/C-modified electrode was more positive than those of the CsPd2/C-, SrPd2/C-, and BaPd2/C-modified electrodes, indicating the presence of more electron-rich Pd NPs in the TBAPd2/C-modified electrode.^62^ Considering that POMs can donate electrons to metal NPs depending on their anion charges, as evidenced by the findings of our previous studies,^59–61^ the observed varying electronic states of Pd NPs likely stem from the differential ability of WO_*x*_ nanospecies to donate electrons to Pd NPs depending on the countercations. However, despite exhibiting a more electron-rich state, Pd NPs derived from the TBAPd2/C-modified electrode displayed poor CO_2_RR performance (Fig. 3a and Table S3†), indicating that the electronic state of Pd NPs does not critically influence the CO_2_RR performance of our system.

Another plausible explanation for the enhanced CO production could be related to the cationic effect. Generally, in the CO_2_RR, electrolytes containing larger cations often demonstrate higher productivity for various CO_2_RR products such as CO.^9–19^ Several theories have been proposed to elucidate the origin of this cationic effect, including (i) modulation of the electric field near the electrode surface, (ii) pH buffering at the electrode interface, and (iii) stabilization of reaction intermediates.^9,64–67^ For instance, Jiang *et al.* recently investigated the CO_2_RR performance of Pd NPs formed on a carbon support using electrolytes containing various alkali metal cations. As anticipated, they observed higher FE_CO_ values for electrolytes containing larger cations, and they attributed these findings to the intensified electric field near the electrode surface.^19^ Although the primary influencing factor in our system remains unidentified at this stage, we propose that the enhanced CO production observed for CsPd2/C, SrPd2/C, and BaPd2/C may be attributed to the abovementioned cationic effect caused by the POM countercations.

## Conclusions

In conclusion, this study highlighted the significant impact of the countercations of POM-based electrocatalysts on their catalytic performance in the CO_2_RR. Specifically, TBAPd2/C, synthesized by immobilizing TBAPd2 on a carbon support, was compared with its analogs CsPd2/C, SrPd2/C, and BaPd2/C, synthesized by exchanging the TBA^+^ cations of TBAPd2/C with alkali metal and alkaline earth metal cations (Cs^+^, Sr^2+^, or Ba^2+^) in acetone. Assessments of their catalytic CO_2_RR performance in a 1 M KHCO_3_ aqueous solution using a flow electrolysis cell with gas-diffusion carbon electrodes modified with these catalysts revealed that the BaPd2/C-modified electrode exhibited selective CO production over 12 h, whereas the TBAPd2/C-modified electrode exhibited lower CO selectivity owing to inadequate suppression of the HER. Detailed analyses indicated that both TBAPd2 and BaPd2 transformed into Pd NPs and WO_*x*_ nanospecies during the CO_2_RR, highlighting the significant influence of countercations on the reactivity of the electrocatalyst. Moreover, Pd(OAc)_2_ demonstrated a marked decline in the CO selectivity during the CO_2_RR, and the formation of larger Pd NPs with a broader size distribution than BaPd2 was observed after the CO_2_RR. These results highlight the critical role of POMs and their countercations in modulating the CO_2_RR performance of POM-based electrocatalysts. As POMs continue to demonstrate promise in CO_2_RR applications, we believe that our findings will contribute to the development of highly effective and durable POM-based electrocatalysts for future CO_2_RR applications.

## Data availability

The data supporting this manuscript is available in the ESI† and available on request.

## Author contributions

K. K. performed the synthesis, reaction, and characterizations. T. Y. performed the XAFS analysis. K. S. conceived and directed the project. All authors analyzed and discussed the results and co-wrote the manuscript.

## Conflicts of interest

There are no conflicts to declare.

## Supplementary Material

SC-OLF-D4SC04304A-s001
