# Supplementary material for: Pd-incorporated polyoxometalate catalysts for electrochemical CO2 reduction
Source: Chem Sci. 2024 Aug 19;15(36):14829–36. doi: 10.1039/d4sc04304a (PMC11342151; doi:10.1039/d4sc04304a)
Supplement: SC-015-D4SC04304A-s001 [file SC-015-D4SC04304A-s001.pdf]

Supplementary Information (SI) for Chemical Science.
This journal is © The Royal Society of Chemistry 2024

Electronic Supplementary Information (ESI) for

Pd-incorporated polyoxometalate catalysts for electrochemical CO_2 reduction

Kimitake Kawakami,[a] Tomohiro Yabe,[a] Fumiaki Amano,[b] Kazuya Yamaguchi[a] and Kosuke Suzuki*[a]

[a] *Department of Applied Chemistry, School of Engineering, The University of Tokyo, 7-3-1 Hongo, Bunkyo-ku, Tokyo 113-8656, Japan. E-mail: ksuzuki@appchem.t.u-tokyo.ac.jp*

[b] *Department of Applied Chemistry for Environment, Graduate School of Urban Environmental Sciences, Tokyo Metropolitan University, 1-1 Minami-Osawa, Hachioji, Tokyo 192-0397, Japan.*

Contents

	Page
1. Experimental Section	S2
2. Supplementary Figures	S7
3. Supplementary Tables	S16
4. References	S21

1. Experimental Section

Instruments

GC analyses were carried out by Nexis GC-2030 gas chromatography (Shimadzu Corporation) equipped with a barrier ionization discharge (BID) detector and a ShinCarbon ST Micropacked column (2.0 m × 1.0 mm I.D., Shinwa Chemical Industries Ltd.). Electrochemical measurements were conducted by VSP-300 multichannel potentiostat (BioLogic). ^{1}H NMR (500.16 MHz) measurements were performed a by JNM ECA-500 spectrometer (JEOL Ltd.) using 5-mm outer-diameter tubes. ^{1}H NMR chemical shifts were referenced to dimethyl sulfoxide signal (2.6 ppm).[S1] IR measurements were carried out by FT/IR-4100 (JASCO Corporation) using KBr disks. ICP-AES measurements were conducted by ICP-8100 (Shimadzu Corporation) and iCAP PRO XP ICP-OES Duo (Thermo Fisher Scientific Inc.) at the Analytical Chemistry Center of the School of Engineering, The University of Tokyo. AAS measurement was performed by ZA3000 (Hitachi High-Tech Corporation) at the Analytical Chemistry Center of the School of Engineering, The University of Tokyo. CHN analyses were carried out by CE-440F Elemental Analyzer (Exeter Analytical Inc.) at the Analytical Chemistry Center of the School of Engineering, The University of Tokyo. TEM observations were conducted by JEM-2010F (JEOL Ltd.). HAADF-STEM observations and STEM-EDS mappings were performed by JEM-ARM200F Thermal FE (JEOL Ltd.).

Materials

Acetone, acetonitrile, dimethyl sulfoxide, D_2O, ethyl acetate, molecular sieve 3A 1/16, phenol, potassium bicarbonate ($KHCO_3$), potassium hydroxide (KOH), sodium chloride (NaCl), and strontium hydroxide octahydrate ($Sr(OH)_2{\cdot}8H_2O$) were obtained from Kanto Chemical Co., Inc. Barium trifluoromethanesulfonate ($Ba(OTf)_2$), trifluoromethanesulfonic acid (HOTf), and 10% tetrabutylammonium hydroxide (TBAOH)-methanol solution were acquired from Tokyo Chemical Industry Co., Ltd. 5% Nafion dispersion was purchased from FUJIFILM Wako Pure Chemical Corporation and Sigma-Aldrich. Palladium acetate ($Pd(OAc)_2$) was obtained from Sigma-Aldrich. All reagent except acetonitrile was used as received. Acetonitrile was dehydrated by using molecular sieve 3A 1/16. Anion exchange membrane (AEM, Fumasep FAB-PK-130) and carbon electrodes (Sigracet 39 BB and AvCarb P75T) were acquired from Fuel Cell Store. AEM was pre-activated by immersing AEM in a 1 M KOH aqueous solution, and then, in a 0.1 M $KHCO_3$ aqueous solution for several days. Carbon dioxide (CO_2, >99.995%) was purchased from TOMOE SHOKAI Co., LTD. **TBAPd2** ($TBA_4[\gamma\text{-}H_2SiW_{10}O_{36}Pd_2(OAc)_2]$)[S2] and cesium trifluoromethanesulfonate (CsOTf)[S3] were synthesized according to the reported procedures.

Synthesis of strontium trifluoromethanesulfonate

Strontium trifluoromethanesulfonate ($Sr(OTf)_2$) was prepared by modifying the reported procedures.[S3,S4] To a solution containing water (10 mL) and HOTf (5 mL, *ca.* 55 mmol), excessive amount of $Sr(OH)_2{\cdot}8H_2O$ (9.21 g, 35 mmol) was added. Then, the mixture was refluxed at 130°C for 1 h. The white precipitate was removed by membrane filtration and the resulting filtrate was evaporated to obtain white crude product. This crude product was dissolved into acetone (20 mL) and insoluble materials were filtered out by syringe filtration. Finally, the resulting filtrate was evaporated to obtain $Sr(OTf)_2$.

Preparation of TBAPd2/C

Immobilization of **TBAPd2** on a carbon support (Vulcan XC 72R) was performed by modifying the reported procedure about immobilization of Keggin-type polyoxometalates (POMs) on single-walled carbon nanotubes.[S5] A carbon support (Vulcan XC 72R, 20 mg) was dispersed in ethyl acetate (25 mL) with the aid of ultrasonication. While vigorously stirring the suspension, an acetone solution (2.5 mL) containing **TBAPd2** (20 mg, 5.3 μmol) was quickly added to the suspension. This mixture was stirred for 1 h and the resultant suspension was stand still for additional 1 h. The black precipitates were collected by membrane filtration, washed with ethyl acetate, and dried under suction to obtain **TBAPd2**/C.

Preparation of CsPd2/C, SrPd2/C, and BaPd2/C

CsPd2/C was prepared by modifying the reported procedure about immobilization of vanadium-incorporated POMs on oxide supports.[S3] **TBAPd2**/C (32 mg) was added to an acetone solution (16 mL) containing CsOTf (4.8 mg, 17 μmol, the cation exchange step), followed by vigorous stirring for 2 h. The black precipitates were collected by membrane filtration and washed with an acetone solution (16 mL) containing CsOTf (2.4 mg, 8.5 μmol, the washing step). Then, the black precipitates were washed with acetone (16 mL) three times and dried under suction to obtain **CsPd2**/C.

SrPd2/C and **BaPd2**/C were prepared by a similar procedure to **CsPd2**/C, using $Sr(OTf)_2$ (3.3 mg, 8.5 μmol) and $Ba(OTf)_2$ (3.7 mg, 8.5 μmol), respectively, for the cation exchange step and $Sr(OTf)_2$ (1.6 mg, 4.3 μmol) and $Ba(OTf)_2$ (1.9 mg, 4.3 μmol), respectively, for the washing step.

Preparation and characterization of BaPd2

An acetone solution (150 mL) containing **TBAPd2** (200 mg, 53.4 μmol) was mixed with an acetone solution (50 mL) containing $Ba(OTf)_2$ (46.5 mg, 107 μmol), followed by vigorous stirring for 2 h. The yellow precipitates were collected by membrane filtration and washed with an acetone solution (200 mL) containing $Ba(OTf)_2$ (23.3 mg, 53.4 μmol). Then, the yellow precipitates were washed with acetone (200 mL) three times and dried under suction. Finally, the yellow precipitates were dried in vacuo to obtain **BaPd2**. IR (KBr pellet, cm^{-1}): 991, 948, 872, 813, 750, 561, 535, 366, 332 (Fig. S2), Elemental analysis calcd (wt%) for $C_7H_{26}Ba_2O_{47}Pd_2SiW_{10}$ ($Ba_2[H_2SiW_{10}O_{36}Pd_2(OAc)_2]\cdot 6H_2O\cdot CH_3COCH_3$): C 2.61, H 0.81, N 0.00, Ba 8.54, Si 0.87, Pd 6.62, W 57.16; found: C 2.36, H 0.92, N 0.02, Ba 8.50, Si 0.89, Pd 6.52, W 57.32.

Preparation of electrodes

The catalyst ink was prepared by ultrasonicating the mixture of catalyst (**TBAPd2**/C: 19.8 mg, **CsPd2**/C: 21.9 mg, **SrPd2**/C: 18.9 mg, or **BaPd2**/C: 20.0 mg), neutralized Nafion solution (200 μL, this solution was prepared by adding 10% TBAOH-methanol solution to 5% Nafion dispersion until the pH reached 7), and acetonitrile (4 mL) for 1 h. For the preparation of the catalyst ink containing $Pd(OAc)_2$, the mixture of a carbon support (10 mg), $Pd(OAc)_2$ (1.12 mg, 4.98 μmol), neutralized Nafion solution (200 μL), and acetonitrile (4 mL) was ultrasonicated for 1 h. The catalyst ink (800 μL) was dropcast onto a carbon electrode (Sigracet 39 BB or AvCarb P75T, 2.5×2.5 cm^2) and the electrode

was dried at 313 K for 3 h. As a carbon electrode, Sigracet 39 BB was used for a 1 h reaction and AvCarb P75T was used for a 12 h reaction.

Electrochemical measurements

Constant potential electrolysis was carried out in a gas-diffusion flow electrolysis cell (Fig. S3) with three-electrode system.[S6,S7] This gas-diffusion flow electrolysis cell is composed of gas chamber for gas delivery and gaseous CO_2RR products collection, catholyte chamber for catholyte circulation and liquid CO_2RR products collection, and anolyte chamber for anolyte circulation. Gas chamber and catholyte chamber were separated by working electrode (WE, catalyst-modified electrode with catalytic area of 1.4×1.4 cm^2) while catholyte chamber and anolyte chamber were separated by pre-activated AEM. Reference electrode (RE, Ag/AgCl electrode filled with a 3 M NaCl aqueous solution, BAS Inc.) and counter electrode (CE, Pt mesh electrode) were placed in catholyte chamber and anolyte chamber, respectively. All potentials were recorded against reversible hydrogen electrode (RHE) using the following equation:

$$E_{\mathrm{RHE}} = E_{\mathrm{Ag/AgCl}} + 0.0591 \times \mathrm{pH} + 0.209 - IR$$

where pH is a pH of the catholyte after the reaction measured by HM-30G (DKK-TOA CORPORATION), I [A] is a current, and R [Ω] is a solution resistance determined by potentiostatic electrochemical impedance spectroscopy. Working electrode, reference electrode, and counter electrode were connected to VSP-300 electrochemical station (BioLogic) to perform electrochemical measurements. A 1 M $KHCO_3$ aqueous solution (30 mL) was used as the catholyte and the anolyte, and they were circulated at 5 mL/min by using a peristaltic pump (TOKYO RIKAKIKAI CO., LTD.). The inlet gas flow rate was controlled at 80 mL/min by 8700MC mass flow controller (KOFLOC Corp.) for CO_2 and 8500MC mass flow controller (KOFLOC Corp.) for Ar. The outlet gas flow rate was measured for each measurement by GFM-2000 flow meter (Shimadzu GLC Ltd.) to quantify gaseous products. Every experiment was conducted under ambient pressure (1 atm) and temperature (298 K). Two independent experiments in each condition were performed.

Carbon monoxide (CO) stripping voltammetry was performed by using the abovementioned gas-diffusion flow electrolysis cell (Fig. S3). Prior to the experiment, the constant potential electrolysis around −0.8 V_{RHE} under CO_2 atmosphere was performed for 10 min in a 1 M $KHCO_3$ aqueous solution. Then, the applied potential was changed to 0.1 V_{RHE} and CO was introduced to the gas chamber for 5 min. Subsequently, while maintaining the applied potential to 0.1 V_{RHE}, the gas chamber and the catholyte were purged with Ar for more than 30 min to remove excess CO. Finally, the potential was linearly swept between 0 V_{RHE} and 1.5 V_{RHE} at a scan rate of 10 mV/s.

Gaseous CO_2RR products analysis

The gaseous CO_2RR products, CO, methane (CH_4), and hydrogen (H_2), were quantified by Nexis GC-2030 gas chromatography (Shimadzu Corporation) equipped with a barrier ionization discharge (BID) detector and a ShinCarbon ST Micropacked column (2.0 m × 1.0 mm I.D., Shinwa Chemical Industries Ltd.). The carrier gas was helium (>99.99995%, TOMOE SHOKAI Co., LTD). The external standard method was used to quantify gaseous CO_2RR products.

The Faradaic efficiency of product i (FE_i) was calculated according to below equation:

$$\mathrm{FE}_i = \frac{n_i F C_i v P}{RTI}$$

where n_i is the number of electrons used to produce one molecule of i, F is the Faraday constant (96485 C/mol), C_i [%] is the concentration of i in the outlet gas, v [m^3/s] is the outlet gas flow rate, P is the atmospheric pressure (1.013×10^5 Pa), R is the universal gas constant (8.314 Pa·m^3/(mol·K)), T is the room temperature (298 K), and I [A] is a current.

Liquid CO_2RR products analysis

The liquid CO_2RR products, formate ($HCOO^-$) and methanol (CH_3OH), were quantified by referring to the previous literature.[S8] The catholyte after the reaction (400 μL) was first mixed with D_2O (200 μL) and internal standard solution (50 μL) containing 20 mM phenol and 10 mM dimethyl sulfoxide. The ^{1}H NMR measurement of this mixture was performed at 303 K using a water suppression method. The concentration of product i in the catholyte (C_i) was determined by using 5-point calibration curve.

The Faradaic efficiency of i (FE_i) was calculated according to below equation:

$$\mathrm{FE}_i = \frac{n_i F C_i V}{Q} \times 100$$

where n_i is the number of electrons used to produce one molecule of i, F is the Faraday constant (96485 C/mol), C_i [mol/L] is the concentration of i in the catholyte, V [L] is the volume of the catholyte (0.030 L), and Q [C] is a total charge passed through the working electrode.

XAFS analysis

XAFS measurements were carried out at the BL14B2 beamline of SPring-8. The X-ray beam was monochromatized using a single pair of Si(311) crystal monochromators for the Pd K-edge and W L_3-edge XAFS. X-ray absorption near-edge structure (XANES) and extended X-ray absorption fine structure (EXAFS) data were analyzed using Athena and Artemis software (Demeter, ver. 0.9.025; Bruce Ravel). The data reduction procedure for EXAFS consisted briefly of the following steps: pre-edge subtraction, background determination, normalization, and spectra averaging. The edge position is defined to be the first inflection point on the leading absorption peak. The energy was calibrated by Pd foil for the Pd K-edge XAFS and W foil for the W L_3-edge XAFS. The background in the EXAFS region was approximated using a cubic spline routine and optimized according to the criteria described by Cook and Sayers.[S9] Then, the spectra were normalized by the edge-step at 50 eV after the absorption edge. The k^3-weighted EXAFS functions were Fourier-transformed, filtered, and fitted in R-space in the range of 3–12 $Å^{-1}$ for Pd and 3–13 $Å^{-1}$ for W. Fourier filtering was used to isolate the contributions of specific shells and to eliminate low frequency background and high frequency noise. Fourier filtering was done by choosing a window in the Fourier-transformed spectrum and calculating the inverse Fourier transform of the selected R-range. The interatomic distance (R), the coordination number (C.N.), the difference of the Debye–Waller factor from the reference (σ^2), and the correction of the threshold energy (ΔE_{j0}) were treated as free parameters during the fitting unless otherwise specified in Table S1. The quality of the fit was estimated from R-factor. R-factor represents the residuals between the observed and calculated spectrum in the fitted range. Low values of R-factor indicate a good agreement between the data and model. To analyze the spectra, simulations of reference compounds using FEFF6 were used to calculate phase shifts and backscattering amplitude. FEFF references were obtained by utilizing crystallographic data of **TPeAPd2** ($TPeA_4[\gamma\text{-}H_2SiW_{10}O_{36}Pd_2(OAc)_2]$, $TPeA^+$ = tetra-*n*-pentylammonium) (crystal system: monoclinic, P 2/c).[S2]

2. Supplementary Figures

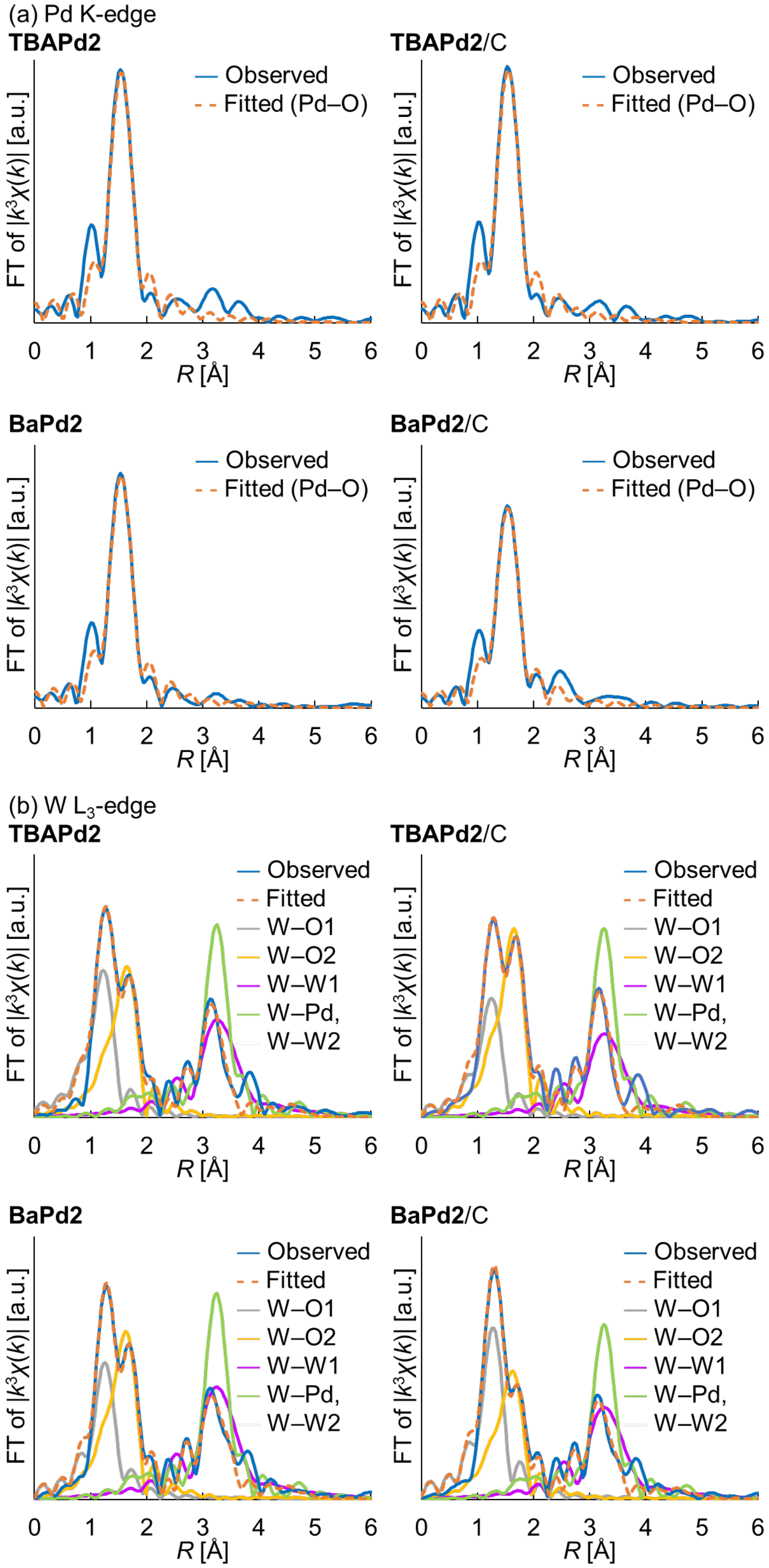

Fig. S1 Fitting results of the Fourier-transformed EXAFS spectra of **TBAPd2**, **TBAPd2**/C, **BaPd2**, and **BaPd2**/C. (a) Pd K-edge. (b) W L_3-edge. Fitting parameters are shown in Table S1.

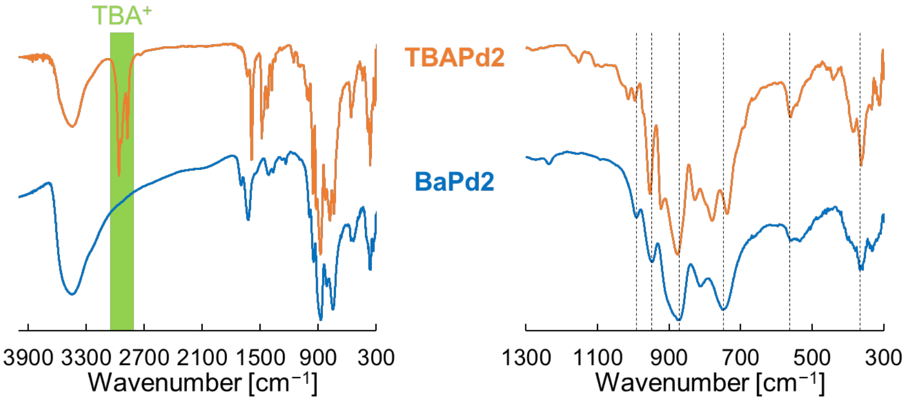

Fig. S2 IR spectra of **TBAPd2** and **BaPd2**.

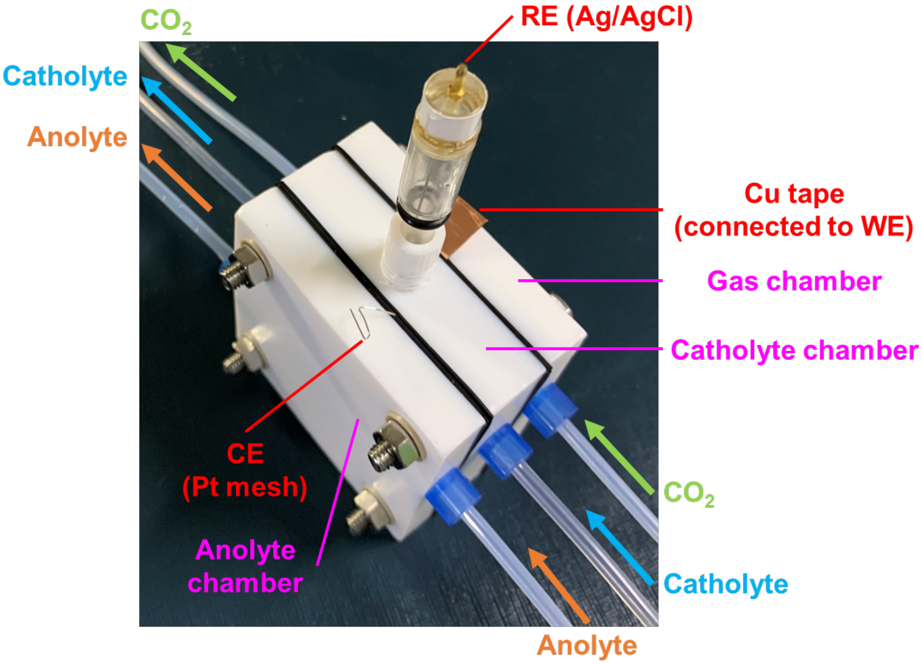

Fig. S3 The configuration of a gas-diffusion flow electrolysis cell for CO_2RR.

(a)

<Chromatogram>

uV

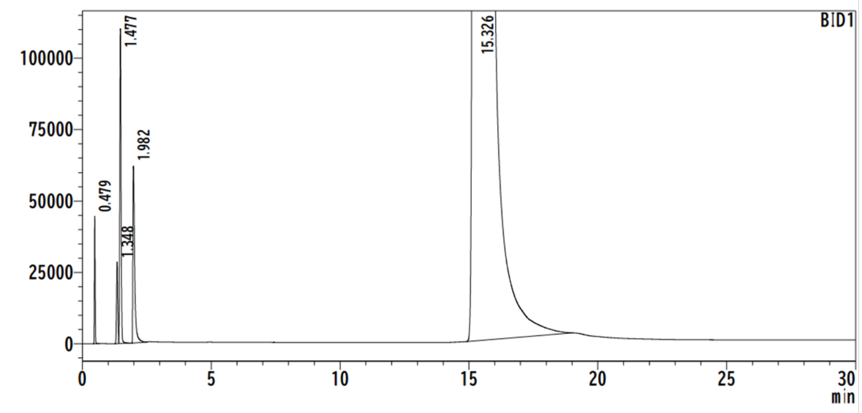

<Peak Table>

BID1

Peak#	Ret. Time	Area	Height	Conc.	Unit	Mark	Name
1	0.479	83925	44416	0.000	ppm		H2
2	1.348	95689	28585	0.000	ppm		O2
3	1.477	395225	110189	0.000	ppm	V	N2
4	1.982	306211	62124	0.000	ppm		CO
5	15.326	114901844	3785946	0.000	ppm		CO2

(b)

<Chromatogram>

uV

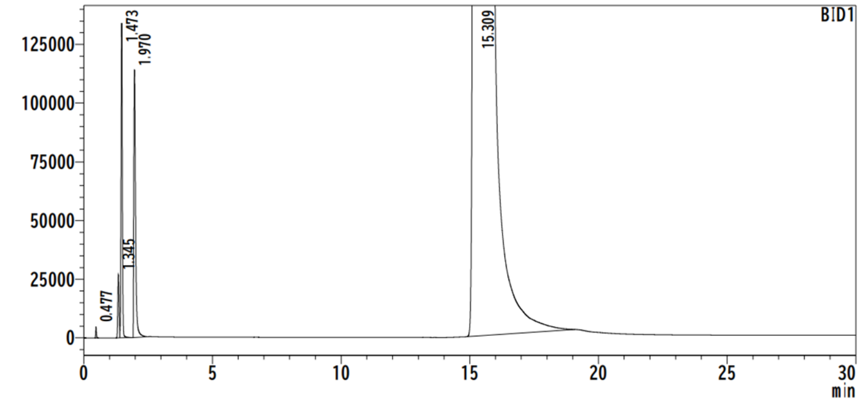

<Peak Table>

BID1

Peak#	Ret. Time	Area	Height	Conc.	Unit	Mark	Name
1	0.477	9033	4745	0.000	ppm		H2
2	1.345	90167	26902	0.000	ppm		O2
3	1.473	478517	133840	0.000	ppm	V	N2
4	1.970	548129	113619	0.000	ppm		CO
5	15.309	114977867	3779147	0.000	ppm		CO2

Fig. S4 GC-BID charts of the outlet gas after a 1 h CO_2RR at −0.80 V_{RHE} using (a) **TBAPd2**/C and (b) **BaPd2**/C.

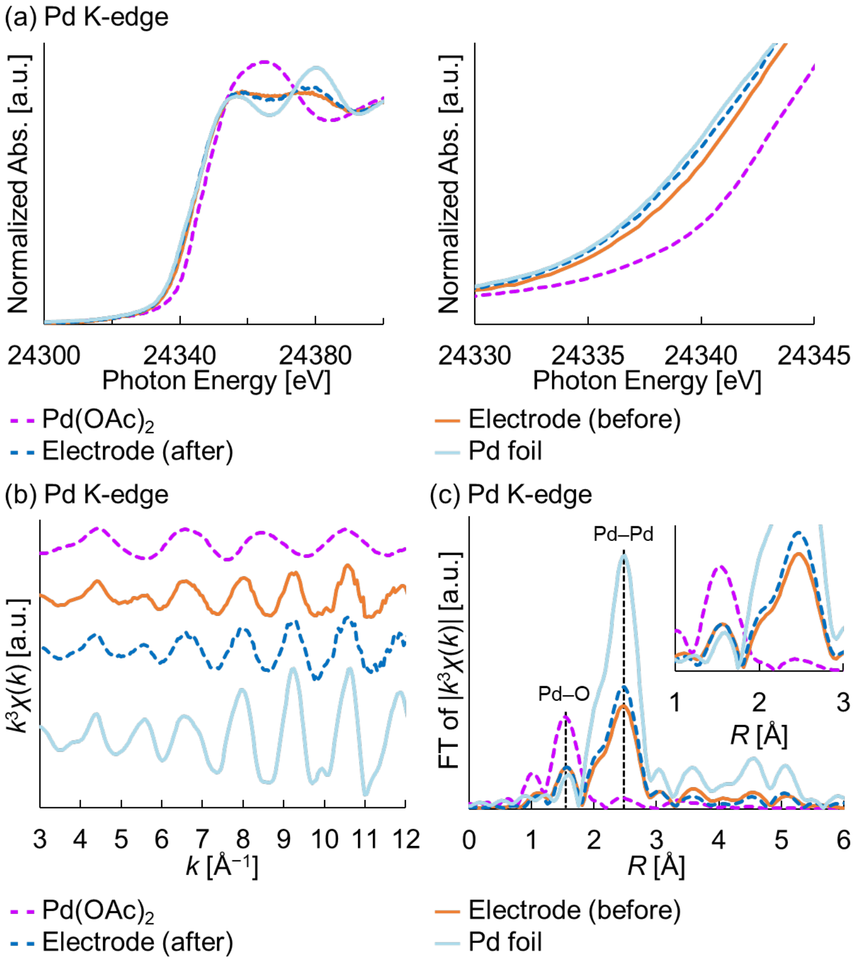

Fig. S5 XAFS characterization of $Pd(OAc)_2$-modified electrode after a 1 h CO_2RR. (a) Pd K-edge XANES spectra. (b) k^3-weighted Pd K-edge EXAFS oscillation (k = 3–12 Å^{-1}). (c) Fourier-transformed Pd K-edge EXAFS spectra.

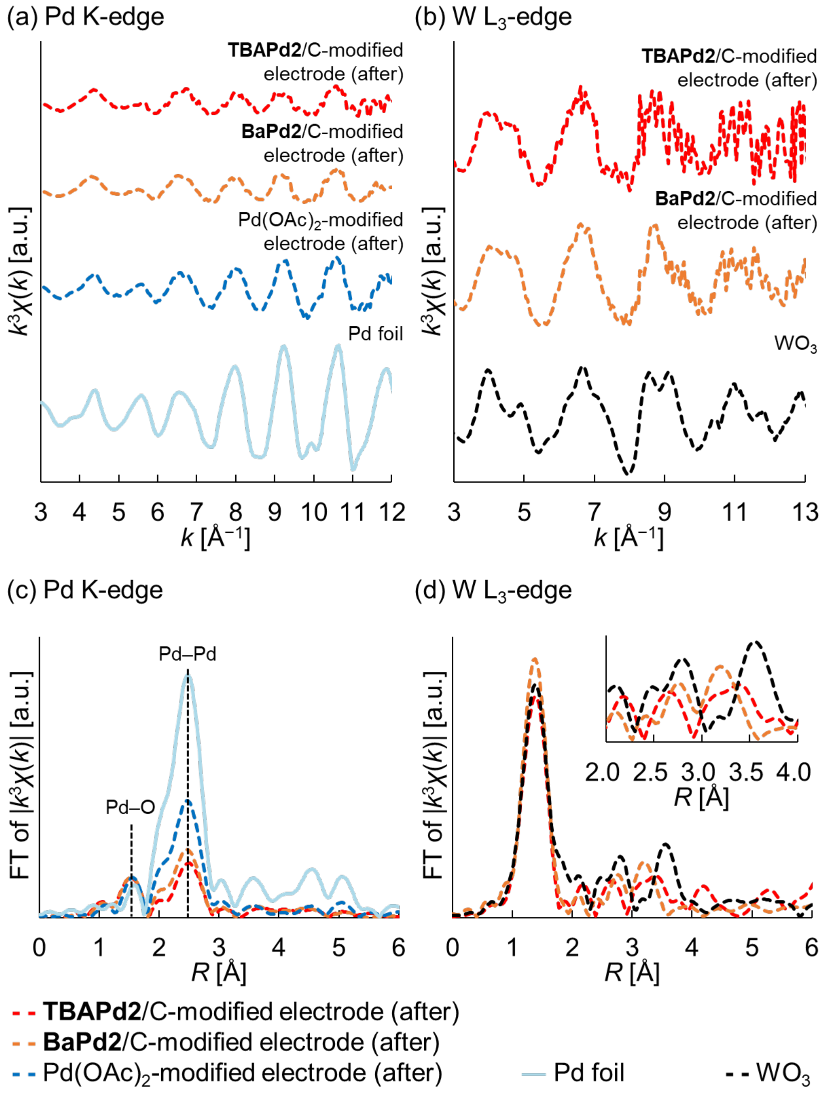

Fig. S6 Comparison of the Pd K-edge and W L_3-edge EXAFS oscillation and Fourier-transformed EXAFS spectra of **TBAPd2**/C-, **BaPd2**/C-, and Pd(OAc)$_2$-modified electrode after a 1 h CO_2RR. (a) k^3-weighted Pd K-edge EXAFS oscillation (k = 3–12 Å^{-1}). (b) k^3-weighted W L_3-edge EXAFS oscillation (k = 3–13 Å^{-1}). (c) Fourier-transformed Pd K-edge EXAFS spectra. (d) Fourier-transformed W L_3-edge EXAFS spectra.

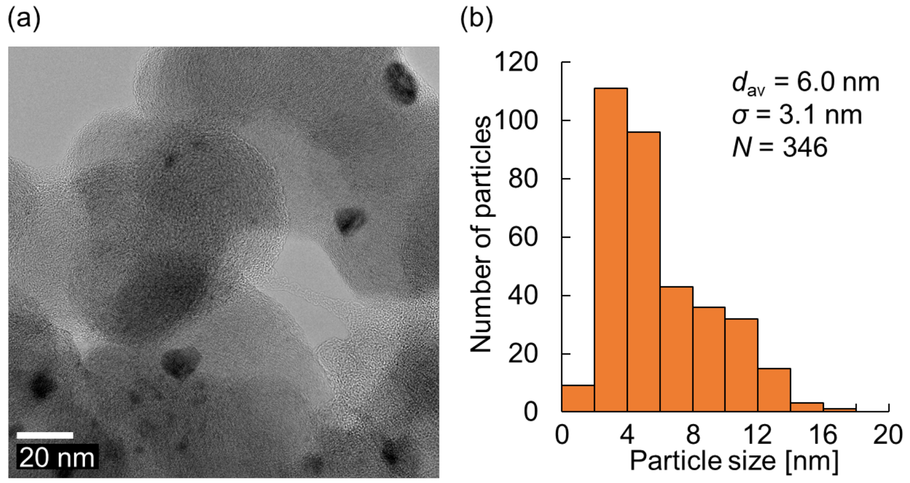

Fig. S7 (a) TEM image of Pd(OAc)$_2$-modified electrode after a 1 h CO_2RR and (b) the corresponding size-distribution histogram.

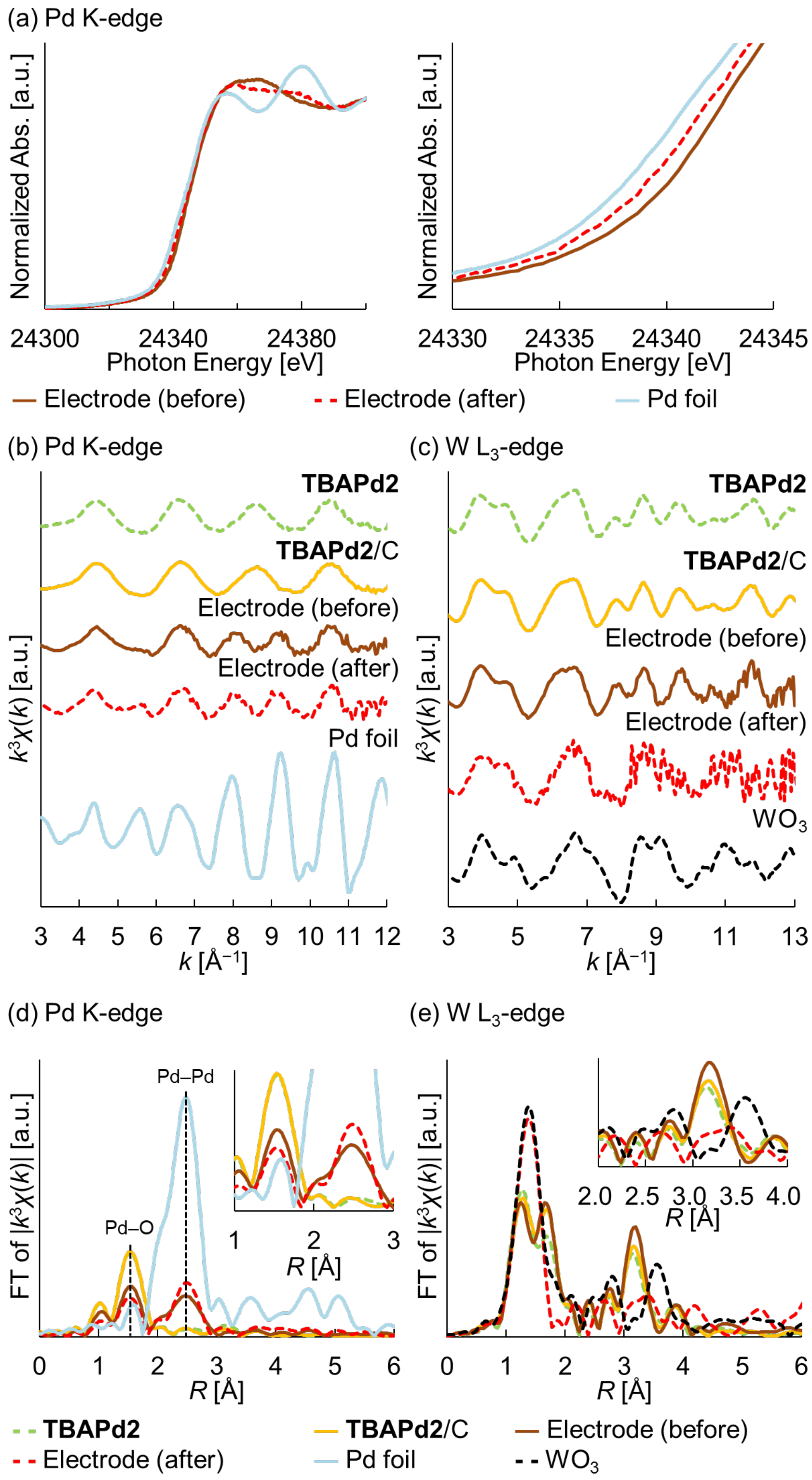

Fig. S8 XAFS characterization of **TBAPd2**/C-modified electrode after a 1 h CO_2RR. (a) Pd K-edge XANES spectra. (b) k^3-weighted Pd K-edge EXAFS oscillation (k = 3–12 Å^{-1}). (c) k^3-weighted W L_3-edge EXAFS oscillation (k = 3–13 Å^{-1}). (d) Fourier-transformed Pd K-edge EXAFS spectra of **TBAPd2**/C-modified electrode after a 1 h reaction. (e) Fourier-transformed W L_3-edge EXAFS spectra.

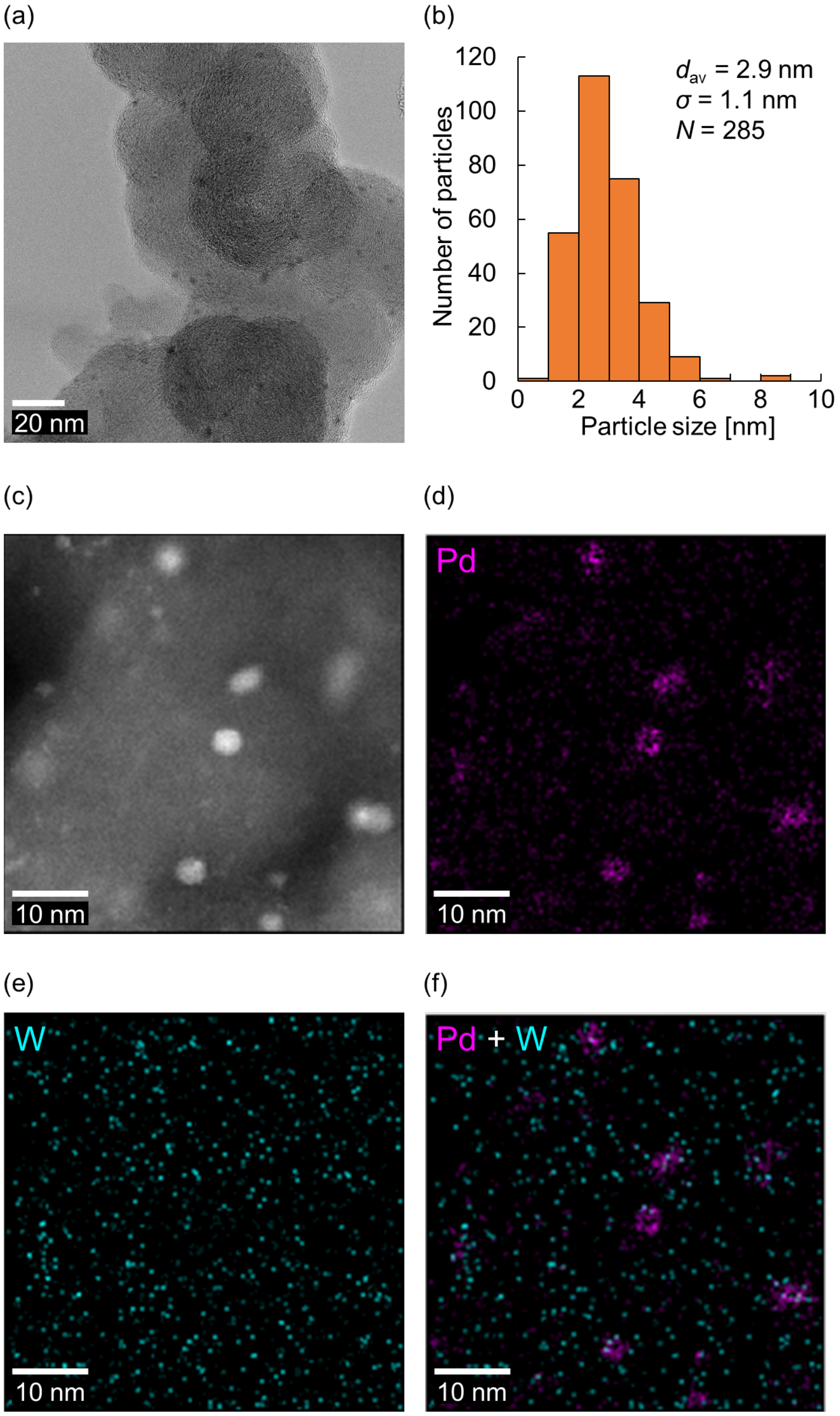

Fig. S9 TEM and HAADF-STEM images of **TBAPd2**/C-modified electrode after a 1 h CO_2RR (a) TEM image and (b) the corresponding size-distribution histogram. (c) HAADF-STEM image and (d–f) the corresponding STEM-EDS mappings (Pd, pink; W, turquoise).

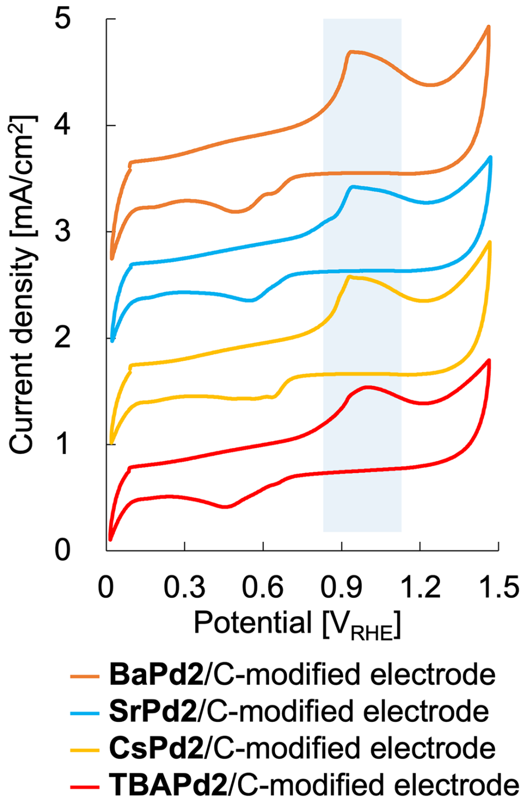

Fig. S10 CO stripping voltammograms of **BaPd2**/C-, **SrPd2**/C-, **CsPd2**/C-, and **TBAPd2**/C-modified electrode after a 10 min CO_2RR in a 1 M $KHCO_3$ aqueous solution (scan rate, 10 mV/s). The region of CO stripping potential is shown in light blue.

3. Supplementary Tables

Table S1 Fitting parameters of EXAFS spectra of **TBAPd2**, **TBAPd2**/C, **BaPd2**, and **BaPd2**/C. (a) Pd K-edge (k = 3–12 Å^{-1}, R = 1.2–2.3 Å). (b) W L_3-edge (k = 3–13 Å^{-1}, R = 1.0–3.6 Å).

(a)

Sample	Shell	C.N.	R [Å]	ΔE_{j0} [eV]	$\sigma^2 \times 10^2$ [Å^2]	R-factor [%]
**TBAPd2**	Pd−O	3.8±0.5	2.00±0.01	2.2±2.0	0.215±0.09	0.6
**TBAPd2**/C	Pd−O	3.7±0.4	2.01±0.01	2.3±2.0	0.201±0.09	0.6
**BaPd2**	Pd−O	3.7±0.4	2.01±0.01	0.5±1.7	0.240±0.08	0.4
**BaPd2**/C	Pd−O	3.2±0.3	2.00±0.01	0.8±1.4	0.255±0.06	0.3

(b)

Sample	Shell	C.N.	R [Å]	ΔE_{j0} [eV]	$\sigma^2 \times 10^2$ [Å^2]	R-factor [%]
**TBAPd2**	W−O1	2.0±0.3	1.70±0.02	−1.1±6.4	0.557 (fixed)	1.6
	W−O2	4.7±0.5	2.18±0.02	6.7±3.3	0.760 (fixed)	
	W−W1	1.5±0.4	3.43±0.01	10.0 (fixed)	0.284 (fixed)	
	W−Pd, W−W2	6.9±1.0	3.67±0.02	5.4±2.3	0.740 (fixed)	
**TBAPd2**/C	W−O1	1.4±0.2	1.70±0.04	3.2±6.8	0.485 (fixed)	1.2
	W−O2	5.4±0.4	2.18±0.05	5.1±2.8	0.676 (fixed)	
	W−W1	1.4±0.4	3.44±0.01	10.0 (fixed)	0.301 (fixed)	
	W−Pd, W−W2	7.2±1.0	3.67±0.03	6.0±2.1	0.772 (fixed)	
**BaPd2**	W−O1	1.4±0.2	1.70±0.02	2.3±7.5	0.367 (fixed)	1.6
	W−O2	5.7±0.5	2.17±0.01	3.6±3.4	0.789 (fixed)	
	W−W1	1.7±0.4	3.42±0.01	10.0 (fixed)	0.284 (fixed)	
	W−Pd, W−W2	7.3±1.0	3.66±0.02	5.8±2.2	0.740 (fixed)	
**BaPd2**/C	W−O1	1.7±0.2	1.72±0.01	7.4±2.6	0.390 (fixed)	1.8
	W−O2	4.2±0.6	2.16±0.02	3.4±5.0	0.764 (fixed)	
	W−W1	1.6±0.4	3.43±0.02	10.0 (fixed)	0.326 (fixed)	
	W−Pd, W−W2	6.8±1.1	3.67±0.02	6.4±2.6	0.787 (fixed)	

Table S2 The results of ICP-AES and AAS measurements of **TBAPd2**/C, **CsPd2**/C, **SrPd2**/C, and **BaPd2**/C.

Sample	wt%			Molar ratio		
	Pd	W	Cs/Sr/Ba	Pd	W	Cs/Sr/Ba
**TBAPd2**/C	2.73	24.65	-	1.91	10	-
**CsPd2**/C	2.42	21.02	6.25	1.99	10	4.11
**SrPd2**/C	2.80	24.52	2.36	1.97	10	2.02
**BaPd2**/C	2.65	23.51	3.40	1.95	10	1.94

Table S3 The results of CO_2RR around −0.8 V_{RHE} for 1 h in 1 M $KHCO_3$ using **TBAPd2**/C, **CsPd2**/C, **SrPd2**/C, **BaPd2**/C, and $Pd(OAc)_2$.

Sample	Potential [V_{RHE}]	Total current density [mA/cm^2]	Faradaic efficiency [%]			
			CO	$HCOO^-$	CH_4	H_2
**TBAPd2**/C	−0.80	27.4±1.1	40.5±7.7	1.9±0.5	n.d.	57.1±1.8
**CsPd2**/C	−0.78	26.8±1.2	72.9±0.1	0.7±0.2	0.2±0.3	21.1±1.7
**SrPd2**/C	−0.78	27.2±0.8	78.9±2.5	0.50±0.01	0.5±0.7	14.8±1.4
**BaPd2**/C	−0.80	24.3±0.6	92.9±4.8	0.35±0.07	n.d.	8.1±1.5
$Pd(OAc)_2$	−0.81	11.66±0.04	83.9±3.6	0.17±0.02	0.5±0.7	10.1±1.1

Table S4 The results of CO_2RR at different potentials for 1 h in 1 M $KHCO_3$ using **BaPd2**/C.

Potential [V_{RHE}]	Total current density [mA/cm^2]	Faradaic efficiency [%]				
		CO	$HCOO^-$	CH_4	CH_3OH	H_2
−0.85	101.2±5.7	50.9±5.5	2.25±0.07	0.3±0.2	<0.1	54.2±1.1
−0.80	24.3±0.6	92.9±4.8	0.35±0.07	n.d.	n.d.	8.1±1.5
−0.70	10.9±0.1	100.5±0.3	0.18±0.04	0.5±0.7	n.d.	2.6±0.3
−0.57	5.0±0.9	90.82±0.05	1.1±0.4	3.6±2.1	n.d.	2.9±0.1

Table S5 The results of control experiments for 1 h in 1 M $KHCO_3$ using a carbon support (C) and **BaPd2**/C.

Sample	Gas	Potential [V_{RHE}]	Total current density [mA/cm^2]	Faradaic efficiency [%]			
				CO	$HCOO^-$	CH_4	H_2
C	CO_2	−0.79	2.9 ± 0.5	n.d.	n.d.	n.d.	103.5 ± 1.2
**BaPd2**/C	Ar	−0.76	33.52 ± 0.07	n.d.	n.d.	n.d.	95.7 ± 0.4
**BaPd2**/C	CO_2	−0.80	24.3 ± 0.6	92.9 ± 4.8	0.35 ± 0.07	n.d.	8.1 ± 1.5

Table S6 The results of CO_2RR at −0.75 V_{RHE} for 12 h in 1 M $KHCO_3$ using **BaPd2**/C.

Time [h]	Total current density [mA/cm^2]	Faradaic efficiency [%]		
		CO	$HCOO^-$	H_2
1	25.8	94.3		11.8
3	29.3	92.5		13.0
6	28.3	90.6	0.3	15.1
9	27.9	90.1		14.8
12	27.8	89.2		16.7

Table S7 The results of CO_2RR at −0.83 V_{RHE} for 12 h in 1 M $KHCO_3$ using $Pd(OAc)_2$.

Time [h]	Total current density [mA/cm^2]	Faradaic efficiency [%]		
		CO	$HCOO^-$	H_2
1	12.3	82.0		25.5
3	13.7	65.3		41.4
6	13.7	57.8	0.7	47.5
9	15.4	51.2		53.1
12	15.2	48.6		55.3

Table S8 The summary of POM-based electrocatalysts for CO_2RR in aqueous electrolytes.

Catalyst	Electrolyte	Potential	FE [%]	Partial current density [mA/cm^2]	Reference
**BaPd2**/C	1 M $KHCO_3$	−0.80 V_{RHE}	92.9±4.8 (CO)	22.5±0.6 (CO)	This work
$[MTRP]^{n+}/[SiW_{12}O_{40}]^{4-}$ (M = Mn^{3+}, Zn^{2+}, Ni^{2+})[a]	0.1 M $NaClO_4$	−0.8 $V_{Ag/AgCl}$	-	-	S10
Co-PMOF[b]	0.5 M $KHCO_3$	−0.8 V_{RHE}	98.7 (CO)	*ca.* 18 (CO)	S11
Bi-PMo nanosheets	0.5 M $NaHCO_3$	−0.86 V_{RHE}	93±2 ($HCOO^-$)	30 ($HCOO^-$)	S12
SiW_{12}-MnL/KB[c]	0.5 M $KHCO_3$	−0.72 V_{RHE}	95 (CO)	*ca.* 14 (CO)	S13
PTC-255[d]	0.1 M $KHCO_3$	−0.8 V_{RHE}	29.4 (CO)	-	S14
Indium sheet	0.1 M Na_2SO_4 containing 2 mM SiW_9V_3	−0.71 V_{RHE}	96.5 (acetate)	*ca.* 0.6 (acetate)	S15
Indium sheet	0.1 M Na_2SO_4 containing 2 mM $SiW_{11}Mn$	−1.0 $V_{Ag/AgCl}$	72.1 (acetate)	*ca.* 0.4 (acetate)	S16
Cu_9S_5 single-unit-cell nanowires	0.1 M $KHCO_3$	−0.8 V_{RHE}	82.0 ($HCOO^-$)	*ca.* 2.5 ($HCOO^-$)	S17
Mo_8@Cu/titanium dioxide nanotube arrays	Saturated $NaHCO_3$	−1.13 V_{RHE}	48.68 (acetate)	56.61 (acetate)	S18
Zn–CoTAPc/PMo12 molecular layer sandwich nanosheets[e]	1 M $KHCO_3$	−0.8 V_{RHE}	97.2 (CO)	108 (CO)	S19
$\{[Cu^{II}_3(tybm)_3(H_2O)_4][\alpha\text{-A-}TeMo_6O_{24}]\}_2\cdot(\alpha\text{-A-}H_6TeMo_6O_{24})\cdot 16H_2O$[f]	0.5 M $KHCO_3$	−0.8 V_{RHE}	93.4 (CO)	-	S20
$[Ag_{11}Na_3(L_1)_4(L_2)_2(H_2O)_4(H_2PMo_9{}^{VI}Mo_3{}^{V}O_{40})_2]$[g]	$KHCO_3$ (concentration not reported)	−0.8 V_{RHE}	97 (CO)	-	S21
$[Zn(MET)_2(H_2O)_2][H_2(\gamma\text{-}Mo_8O_{26})]$[h]	0.5 M $KHCO_3$	−0.8 V_{RHE}	92.8 (CO)	-	S22

Table S8 (continued) The summary of POM-based electrocatalysts for CO_2RR in aqueous electrolytes.

Catalyst	Electrolyte	Potential	FE [%]	Partial current density [mA/cm^2]	Reference
Au−PW_{12} sub-1 nm nanowires	0.1 M $KHCO_3$	−0.9 V_{RHE}	90.64 (CO)	-	S23
H-POM@PCN-222(Co)[i]	0.5 M $KHCO_3$	−0.8 V_{RHE}	96.2 (CO)	*ca.* 11 (CO)	S24
Fe-POMOF[j]	0.5 M $KHCO_3$	−0.7 V_{RHE}	92.1 (CO)	3.2 (CO)	S25
**{$Ag_{49}Mo_{16}$}**[k]	0.5 M $KHCO_3$	−0.8 V_{RHE}	44.75 (CO)	*ca.* 4 (CO)	S26
$P_2W_{18}Mn_4$@PCN-222	0.5 M $KHCO_3$	−0.60 V_{RHE}	72.6 (CO)	*ca.* 0.3 (CO)	S27
$Cu_4[\alpha_2$-$P_2W_{17}O_{61}In(OH)]$-decorated indium hydroxide nanocrystals	0.1 M $KHCO_3$	−0.6 V_{RHE}	*ca.* 10 (CO)	-	S28
Indium sheet	0.1 M Na_2SO_4 containing 2 mM PV_3Mo_9	−0.3 V_{RHE}	93.4 (ethanol)	-	S29
H-SiW_{11}Co@PCN-224	0.5 M $KHCO_3$	−0.80 V_{RHE}	89.9 (CO)	*ca.* 5 (CO)	S30
Indium sheet	0.1 M Na_2SO_4 containing 2 mM $PVMoW_{10}$	−1.0 $V_{Ag/AgCl}$	75.6 (acetate)	-	S31
Indium sheet	0.1 M Na_2SO_4 containing 2 mM PV_2MoW_9	−0.7 $V_{Ag/AgCl}$	85.1 (ethanol)	-	S31

a TRP = μ-(meso-5,10,15,20-tetra(pirydil)porphyrin)tetrakis{bis(bipyridine)chloride ruthenium(II)}
b Co-PMOF = $[PMo^{V}_8Mo^{VI}_4O_{35}(OH)_5Zn_4]_2$[Co-TCPP]$[2H_2O]$[1.5TBAOH], TCPP = tetrakis[4-carboxyphenyl]-porphyrin
c SiW_{12}-MnL = $[Mn^{I}(bipy)(CO)_3(CH_3CN)]_4(SiW_{12}O_{40})\cdot 5CH_3CN$, KB = Ketjen black
d PTC-255 = $Ag_2Ti_{12}(\mu_3\text{-}O)_{12}(\mu_2\text{-}O)_2(4\text{-FBA})_{14}(\mu_2\text{-OMe})_4(OMe)_4\cdot 2CH_3CN$, 4-FBA = 4-fluorobenzoate
e CoTAPc = cobalt tetraaminophthalocyanine
f tybm = 4-[1,3,4]triazol-4-yl-benzylamine
g L_1 = 1H-1,2,3,5,10-pentaaza-cylopenta[b]anthracene-4,11-dione, L_2 = 6-nitro-1H-benzotriazole
h MET = 4-(3-imidazol-1-yl-ethyl)-4*H*-[1,3,4]triazole
i POM = $Co^{III}Co^{II}(H_2O)W_{11}O_{39}$
j Fe-POMOF = $[PMo^{V}_8Mo^{VI}_4O_{35}(OH)_5Zn^{II}_4]_2[Fe^{III}\text{-TCPP-Cl}]\cdot$Guest, TCPP = tetrakis(4-carboxyphenyl)-porphyrin
k **{$Ag_{49}Mo_{16}$}** = $[(Mo_6O_{22})@H_3Ag_{49}(MoO_3)_9(MoO_4)(TC4A)_6(^{i}PrS)_{18}(CH_3CN)_2(H_2O)]$, TC4A = thiacalix[4]arene

4. References

S1 K. P. Kuhl, E. R. Cave, D. N. Abramc and T. F. Jaramillo, *Energy Environ. Sci.*, 2012, **5**, 7050.

S2 T. Hirano, K. Uehara, K. Kamata, and N. Mizuno, *J. Am. Chem. Soc.*, 2012, **134**, 6425.

S3 T. Suzuki, T. Yabe, K. Wachi, K. Yonesato, K. Suzuki and K. Yamaguchi, *ChemNanoMat*, 2023, **9**, e202200428.

S4 J. H. Forsberg, V. T. Spaziano, T. M. Balasubramanian, G. K. Liu, S. A. Kinsley, C. A. Duckworth, J. J. Poteruca, P. S. Brown and J. L. Miller, *J. Org. Chem.*, 1987, **52**, 1017.

S5 H. Wang, N. Kawasaki, T. Yokoyama, H. Yoshikawa and K. Awaga, *Dalton Trans.*, 2012, **41**, 9863.

S6 K. Liu, W. A. Smith and T. Burdyny, *ACS Energy Lett.*, 2019, **4**, 639.

S7 H.-P. Iglesias van Montfort, S. Subramanian, E. Irtem, M. Sassenburg, M. Li, J. Kok, J. Middelkoop and T. Burdyny, *ACS Energy Lett.*, 2023, **8**, 4156.

S8 S. Gonglach, S. Paul, M. Haas, F. Pillwein, S. S. Sreejith, S. Barman, R. De, S. Müllegger, P. Gerschel, U.-P. Apfel, H. Coskun, A. Aljabour, P. Stadler, W. Schöfberger and S. Roy, *Nat. Commun.*, 2019, **10**, 3864.

S9 J. W. Cook Jr. and D. E. Sayers, *J. Appl. Phys.*, 1981, **52**, 5024.

S10 M. García, M. J. Aguirre, G. Canzi, C. P. Kubiak, M. Ohlbaum and M. Isaacs, *Electrochim. Acta*, 2014, **115**, 146.

S11 Y.-R. Wang, Q. Huang, C.-T. He, Y. Chen, J. Liu, F.-C. Shen and Y.-Q. Lan, *Nat. Commun.*, 2018, **9**, 4466.

S12 S.-X. Guo, Y. Zhang, X. Zhang, C. D. Easton, D. R. MacFarlane and J. Zhang, *ChemSusChem*, 2019, **12**, 1091.

S13 J. Du, Z.-L. Lang, Y.-Y. Ma, H.-Q. Tan, B.-L. Liu, Y.-H. Wang, Z.-H. Kang and Y.-G. Li, *Chem. Sci.*, 2020, **11**, 3007.

S14 Y.-J. Liu, P. Shao, M.-Y. Gao, W.-H. Fang and J. Zhang, *Inorg. Chem.*, 2020, **59**, 11442.

S15 B. Zha, C. Li and J. Li, *J. Catal.*, 2020, **382**, 69.

S16 C. Li, B. Zha and J. Li, *J. CO2 Util.*, 2020, **38**, 299.

S17 D. Yang, S. Zuo, H. Yang and X. Wang, *Adv. Energy Mater.*, 2021, **11**, 2100272.

S18 D. Zang, Q. Li, G. Dai, M. Zeng, Y. Huang and Y. Wei, *Appl. Catal. B*, 2021, **281**, 119426.

S19 H. Yang, D. Yang, Y. Zhou and X. Wan, *J. Am. Chem. Soc.*, 2021, **143**, 13721.

S20 J. Ying, B. Zhang, A. Tian and X. Wang, *CrystEngComm*, 2021, **23**, 2572.

S21 H. Yu, J. Ying, Y. Tian and A. Tian, *Inorg. Chem. Commun.*, 2021, **123**, 108338.

S22 B. Zhang, J. Ying, X. Zhang, C. Wang and A. Tian, *New J. Chem.*, 2021, **45**, 13340.

S23 J. Liu, S. Wang, N. Liu, D. Yang, H. Wang, H. Hu, J. Zhuang and X. Wang, *Small*, 2021, **17**, 2006260.

S24 M.-L. Sun, Y.-R. Wang, W.-W. He, R.-L. Zhong, Q.-Z. Liu, S. Xu, J.-M. Xu, X.-L. Han, X. Ge, S.-L. Li, Y.-Q. Lan, A. M. Al-Enizi, A. Nafady and S. Ma, *Small*, 2021, **17**, 2100762.

S25 Q. Huang, Q. Niu, X.-F. Li, J. Liu, S.-N. Sun, L.-Z. Dong, S.-L. Li, Y.-P. Cai and Y.-Q. Lan, *Sci. Adv.*, 2022, **8**, eadd5598.

S26 S.-Q. Li, L.-F. Dai, Y.-Q. Tian, Y.-X. Yi, J. Yan and C. Liu, *Chem. Commun.*, 2023, **59**, 575.

S27 M.-T. Peng, C. Chen, Y. Zhang, J.-Y. Xu, Y.-L. Teng and B.-X. Dong, *Dalton Trans.*, 2023, **52**, 10737.

S28 T. Tubul-Sterin, M. Baranov, G. Gan-Or, N. Leffler, A. Neyman and I. A. Weinstock, *Inorg. Chem.*, 2023, **62**, 1804.

S29 W. Sun, D. Yao, Y. Tai, L. Zhou, W. Tian, M. Yang and C. Li, *J. Colloid Interface Sci.*, 2023, **650**, 121.

S30 Y. Zhang, C. Li, L. Shu, Y.-L. Teng and B.-X. Dong, *Inorg. Chem.*, 2024, **63**, 11592.

S31 Y. Tai, W. Sun, D. Yao, L. Zhou, W. Tian, H. Yan and C. Li, *Polyoxometalates*, 2024, **3**, 9140051.
